# Maternal ontogeny modulates auxin-associated cutting mortality in *Amentotaxus formosana*, with genotype-specific responses revealed by Cox proportional hazards modelling

**DOI:** 10.3389/fpls.2026.1892264

**Published:** 2026-08-19

**Authors:** Ya-Zhu Ko, Huie-Chuan Shih, Meng-Shin Shiao, Tsai-Wen Hsu, Yu-Chung Chiang

**Affiliations:** 1 Department of Biological Sciences, National Sun Yat-sen University, Kaohsiung, Taiwan; 2 Department of Nursing, Meiho University, Pingtung, Taiwan; 3 Research Laboratory Section, Offices of Health Science Research, Faculty of Medicine Ramathibodi Hospital, Mahidol University, Bangkok, Thailand; 4 Taiwan Biodiversity Research Institute, Nantou, Taiwan; 5 Department of Biomedical Science and Environment Biology, Kaohsiung Medical University, Kaohsiung, Taiwan

**Keywords:** *Amentotaxus formosana*, ex situ conservation, genotype × environment interaction, survival analysis, Taxaceae, vegetative propagation

## Abstract

Amentotaxus formosana (Taxaceae) is an endangered relict conifer for which stem-cutting propagation is essential for *ex situ* conservation. Seed-based regeneration is unreliable in its small, fragmented, dioecious populations. However, extreme and uncharacterized cutting mortality has severely bottlenecked these efforts. Because conventional static endpoint evaluations mask the critical temporal dynamics of rhizogenesis and failure, the underlying drivers of this attrition remain obscure. Here, we applied Kaplan-Meier estimation and Cox proportional hazards modelling to a two-stage factorial experiment involving 641 cuttings to identify factors associated with cutting mortality over time. Stage 1 revealed that substrate composition significantly influenced survival, with pure shredded shale approximately doubling the mortality hazard relative to peat-perlite. Among plant growth regulator (PGR) treatments, indole-3-butyric acid (IBA) significantly reduced mortality risk relative to the ABT-1 composite treatment. Stage 2 identified specific maternal-DBH- and genotype-dependent treatment responses within an analytical subset of five adequately sampled genotypes. The association between maternal DBH and mortality hazard was treatment-dependent, with a significant maternal DBH × IBA interaction. In analyses including all 12 genotypes, stratified log-rank tests detected significant among-genotype survival differences under IBA and NAA, but not under the untreated control. Within the five-genotype Cox model, DG-15–12 showed a significant genotype × NAA interaction. This study represents the first application of time-to-event survival analysis in *Amentotaxus* propagation. We demonstrate that cutting mortality is partially associated with interactions involving substrate type, auxin treatment, maternal DBH, and genotype. However, the magnitude and statistical detectability of these interactions varied across factor combinations. These findings provide a quantitative framework for genotype-resolved, risk-informed conservation propagation of recalcitrant relict species.

## 1 Introduction

*Amentotaxus formosana* H.L. Li (Taxaceae) is a dioecious, slow-growing relict tree endemic to the southernmost ridges of Taiwan’s Central Mountain Range. It persists in a few geographically isolated montane localities between 700 and 1,500 m elevation, principally within the Dawu Taiwan Amentotaxus Nature Reserve, Chachayalaishan Major Wildlife Habitat, and Dalili Mountain (Yang, 2007; Ko et al., 2025). To protect these core habitats against anthropogenic disturbance, the species is afforded strict statutory protection as a Natural Monument under the Cultural Heritage Preservation Act, with its primary sites mandated for *in situ* conservation (Yang, 2007; Ko et al., 2025). The species faces a severe extinction threat owing to its extremely narrow distribution, small population size, and severely limited sexual reproductive capacity, as reflected in its formal designation as Endangered (EN) in both the 2017 Red List of Vascular Plants of Taiwan (Editorial Committee of the Red List of Taiwan Plants, 2017; Ko et al., 2025) and the IUCN Red List assessment (Chiang, 2023).

The precarious status of *A. formosana* is compounded by severe reproductive and genetic constraints. Its dioecious breeding system and spatial isolation restrict effective pollen transfer, while its heavy seeds exhibit deep dormancy and produce few viable offspring. Consequently, natural regeneration relies heavily on basal clonal sprouting, a strategy that maintains local cover without contributing new genetic diversity or facilitating inter-population connectivity (Yang, 2007; Chung et al., 2019; Ko et al., 2025). Indeed, recent microsatellite analysis across the three main populations confirmed that within-population genetic diversity is critically low, inbreeding is severe, and effective population sizes are in sustained decline (Ko et al., 2025). Given these limitations, stem-cutting propagation has emerged as the most practical vehicle for *ex situ* conservation, as it preserves donor genotype fidelity and circumvents seed dependency. Yet despite this recognized necessity, the only published propagation trial conducted specifically on *A. formosana* found that over 90% of initially rooted cuttings died within two years of nursery cultivation (Chung et al., 2019). Thus, the species currently lacks a viable, long-term clonal multiplication protocol.

The persistence of this propagation failure reflects two biological constraints that are particularly acute in long-lived Taxaceae and remain poorly characterized for *A. formosana*. The first is ontogenetic aging of the donor plant. As trees mature, the capacity of shoot tissues to initiate adventitious roots declines progressively, through a cascade of changes that includes falling endogenous indole-3-acetic acid (IAA) biosynthesis, rising abscisic acid concentrations, reduced expression of auxin receptor genes (TIR1, TAA1), and the accumulation of phenylpropanoid compounds that drive callus lignification and form physical barriers against rhizogenesis (Álvarez et al., 2016; Aumond et al., 2017; Chang et al., 2023). In *Taxus baccata*, the most extensively studied Taxaceae species, this ontogenetic decline reduces rooting success from approximately 44% in juvenile donors to only 8–20% in reproductively mature trees (Savev and Iliev, 2023). For *A. formosana*, the practical relevance of this gradient is further complicated by the difficulty of estimating donor age directly. The species’ characteristic basal-sprouting growth habit obscures the relationship between morphological appearance and chronological age, making diameter at breast height (DBH) the most accessible field proxy for determining ontogenetic stages in conservation practice (Lin et al., 2007; Ho et al., 2012; Chung et al., 2019).

The second constraint is the substantial intraspecific variation in auxin responsiveness among genotypes. Across a broad range of woody plant and rare tree taxa, rooting success under identical auxin treatments varies enormously among clones of the same species, with genotype-by-auxin interactions that are both statistically significant and practically consequential (Gera et al., 2001; Nakhooda et al., 2014; Dhiman and Gandhi, 2017; Martínez et al., 2020, 2021). In *Eucalyptus grandis* × *E. nitens*, for instance, increasing IBA concentration from 0.5 to 8.0 ag kg^-^¹ reduced survival in clone 1 from 70% to 6%, while clone 3 under the same concentration gradient showed a modest improvement from 75% to 80% (Nakhooda et al., 2014). Statistically significant genotype-by-IBA interactions have similarly been reported in *Quercus ilex*, where different genotypes responded optimally to treatments of different concentration and duration (Martínez et al., 2020), and in *Triplochiton scleroxylon*, where some clones achieved near-complete rooting at the softwood stage while others remained recalcitrant regardless of treatment (Leakey et al., 1982).

In *A. formosana*, a similar pattern is already apparent from the limited data available: rooting rates among 61 wild donor trees varied from 13% to 100% under the same propagation conditions (Chung et al., 2019). Despite this clear indication of substantial genotypic variation, detailed experimental evaluations addressing how specific *A. formosana* genotypes interact with varying auxin treatments and substrate compositions remain entirely absent. Characterizing these genotype-specific responses is therefore a necessary prerequisite for developing effective, scalable clonal propagation strategies for this endangered species.

To address these knowledge gaps, this study investigates the complex, previously uncharacterized dynamics among ontogenetic stage, genotype identity, substrate composition, and exogenous auxin treatments. Traditional single-endpoint analyses are methodologically insufficient to capture the temporal dynamics of cutting mortality and rhizogenesis in slow-growing species. Therefore, we employed a time-to-event analytical framework, coupling Kaplan-Meier estimation with Cox proportional hazards modelling to track these processes over time, testing two core biological hypotheses: First, that increasing maternal DBH will be associated with higher cutting mortality hazard under at least some exogenous auxin treatments, producing a significant DBH × PGR interaction. Second, that the effects of standardized high-concentration auxin treatments on cutting survival will not be uniform across genotypes but will instead express genotype-by-auxin interactions of varying magnitude. By resolving these hypotheses through dynamic survival modelling, this study aims to shift propagation evaluation from endpoint assessment to risk-informed temporal analysis, providing the foundation for genotype-resolved *ex situ* conservation protocols of recalcitrant relict taxa.

## 2 Materials and methods

### 2.1 Plant material, study site selection, and experimental design

All stem cuttings were collected from donor trees in the Dawu Taiwan Amentotaxus Nature Reserve (DAWU), the only *A. formosana* population with a complete individual-level ecological census (**Supplementary Table 1**) and with safe, repeatable field access for cutting harvest. The full rationale for restricting sampling to DAWU is given in **Supplementary Methods S1.1**. Donor trees were pre-screened with a five-tier health scale (Forestry Management Office, Pingtung Forest District, 2009; Ho et al., 2012) (**Supplementary Table 1**), and only trees scoring 3 or above were collected, with the healthiest grades (4 and 5) preferred where sample sizes within a management unit permitted. The tier definitions and the reasoning behind the grade 3 threshold are given in **Supplementary Methods S1.2**.

Donor genotypes were assigned to 15 fine-scale management units (MUs; DG-15–1 through DG-15-15) previously delineated by Bayesian spatial clustering of microsatellite loci within the DAWU population (Ko et al., 2025) (**Supplementary Table 1**). Of the 15 management units, 12 were represented in the cutting experiment (DG-15–1 through DG-15-4, DG-15-6, DG-15-7, DG-15-9, and DG-15–11 through DG-15-15). Three management units DG-15-5, DG-15-8, and DG-15–10 could not be included because all individuals assigned to these units scored 1 or 2 on the health index at the time of harvest, indicating severely compromised physiological condition. A total of 33 donor trees representing the 12 eligible management units were selected for Stage 2 (**Supplementary Table 1**).

The 33 sampled donor trees ranged in DBH from 1.2 to 30.0 cm (**Supplementary Table 1**), spanning the full continuum from juvenile individuals through middle-aged trees to fully mature stages. To evaluate the effect of maternal ontogenetic stage on cutting survival and plant growth regulator (PGR) sensitivity without compromising the statistical power of the Cox proportional hazards model, donors were stratified at the median DBH of the 33 donor trees, 7.5 cm. This statistical cut-point also corresponds to the midpoint of the middle-aged range (5 to 10 cm) previously described for the species (Chan, 2005), giving a Small DBH group (DBH < 7.5 cm) and a Large DBH group (DBH ≥ 7.5 cm) that capture relative ontogenetic status rather than chronological age. DBH was used both as a categorical group for Kaplan-Meier visualisation and as a continuous covariate in the Cox model (Royston et al., 2006).

The experimental framework was organized into two sequential factorial trials, structured to transition from broad treatment screening to genotype-resolved survival analysis. In Stage 1, we evaluated the main effects and interactions of substrate and PGR treatments across the collective Dawu population to establish an empirical baseline for Stage 2. Stage 2 was implemented at the individual-genotype level to examine whether the association between maternal DBH and mortality differed among auxin treatments.

For Stage 1 (26 September 2013 to 24 February 2015), apical shoot cuttings measuring approximately 10–25 cm in length and retaining their full complement of needle leaves were harvested for the propagation trials. The same harvesting specification was applied in Stage 2. Length was set as a specification for harvesting rather than measured for each cutting, so individual cutting length was not recorded and is not available as a covariate in any analysis reported here. Cuttings were assigned to one of five PGR treatments. The control group (CK) consisted of an untreated water dip. Three pure exogenous auxins were evaluated using a high-concentration quick-dip method, in which the basal 2 cm of each cutting was immersed in a 2000 ppm solution for 30 seconds. These auxins included indole-3-acetic acid (IAA, ≥98%; Alfa Aesar, Ward Hill, MA, USA), indole-3-butyric acid (IBA, 98%; Acros Organics, Geel, Belgium), and 1-naphthaleneacetic acid (NAA, >98%; Tokyo Chemical Industry Co., Ltd., Tokyo, Japan). Finally, the ABT-1 composite rooting powder (ABT; containing 30% indole-3-acetic acid and 20% 1-naphthaleneacetic acid; Beijing ABT Research and Development Centre, Beijing, China) was applied via a low-concentration prolonged-soak method, with the basal 2 cm immersed in a 200 ppm solution for two hours (Wang, 1989; Chen, 2006).

Five distinct rooting substrates were evaluated. Substrate A was a 2:1 (v/v) mix of peat moss and perlite, referred to hereafter as peat-perlite. Substrate B was a 2:2:1:1 (v/v) mix of shredded shale, akadama soil, carbonised rice husk, and perlite, hereafter shale-akadama mix. Substrate C was a 3:1 (v/v) mix of coconut fibre and carbonised rice husk, hereafter coconut-fibre mix. Substrate D was pure shredded shale (**Supplementary Figure 1A**). Substrate E was pre-moistened horticultural rockwool blocks into which cuttings were directly inserted for continuous hydration, hereafter rockwool (**Supplementary Figure 1**). Shredded shale was incorporated into Substrates B and D to emulate the edaphic conditions of *A. formosana*’s native microhabitat. Vegetation surveys indicate that natural populations are predominantly concentrated on upper- to mid-slopes and along the margins of colluvial gullies, where the rooting zone is defined by exceptional stoniness and a substantial proportion of coarse lithic material (Yang, 2007; Ho et al., 2012). Per-combination sample sizes varied from 7 to 20 cuttings, reflecting the unequal availability of shoot material across donor trees, giving a Stage 1 total of 252 cuttings (**Table 1**).

**Table 1:** Experimental design and sample size distribution for the two-stage propagation experiments.

Trial stage	Experimental period	Experimental factors	Main condition	Breakdown of sub-treatments (n)	Total N
Stage 1	2013.09.26 – 2015.02.24	PGR & Substrate	CK (Control)	Substrate A (7), B (9), C (8), D (17), E (7)	252
			ABT	Substrate A (8), B (8), C (8), D (15), E (11)	
			NAA	Substrate A (8), B (8), C (7), D (12), E (11)	
			IBA	Substrate A (8), B (9), C (8), D (20), E (11)	
			IAA	Substrate A (8), B (8), C (8), D (14), E (14)	
Stage 2	2014.02.15 – 2015.02.24	Genotype & PGR	DG-15-6	CK (12), IBA (20), NAA (20)	389
			DG-15-11	CK (7), IBA (15), NAA (16)	
			DG-15-15	CK (8), IBA (15), NAA (15)	
			DG-15-12	CK (6), IBA (16), NAA (15)	
			DG-15-13	CK (7), IBA (15), NAA (14)	
			DG-15-7	CK (5), IBA (15), NAA (14)	
			DG-15-2	CK (6), IBA (13), NAA (14)	
			DG-15-4	CK (4), IBA (12), NAA (14)	
			DG-15-3	CK (6), IBA (9), NAA (9)	
			DG-15-9	CK (3), IBA (12), NAA (9)	
			DG-15-14	CK (7), IBA (7), NAA (8)	
			DG-15-1	CK (4), IBA (9), NAA (8)	

[Substrate definitions] Substrate codes are A (peat-perlite), B (shale-akadama mix), C (coconut-fibre mix), D (pure shredded shale), and E (rockwool).

[Treatment definitions] CK: Control group (untreated). ABT: ABT-1 rooting powder (200 ppm). IAA: Indole-3-acetic acid (2000 ppm). IBA: Indole-3-butyric acid (2000 ppm). NAA: 1-Naphthaleneacetic acid (2000 ppm).

For Stage 2 (15 February 2014 to 24 February 2015), cuttings from the 12 selected *A. formosana* genotypes were propagated using Substrate A. This medium was selected from Stage 1 because it showed the most stable and generally highest survival profile across PGR treatments, thereby providing a comparatively low-risk hydrological background for evaluating genotype-specific PGR responses. Incorporating multiple substrates into the Stage 2 factorial design was not feasible given the severely limited availability of wild-collected shoot material from individual donor trees (**Table 1**). Accordingly, cuttings were subjected to three rooting treatments: an untreated control (CK), 2000 ppm IBA, and 2000 ppm NAA, all applied via a 30-second quick-dip method. Because per-genotype sample sizes were strictly constrained by the natural availability of suitable shoots on wild donors (**Table 1**), we preferentially allocated material to the auxin treatments (IBA = 158, NAA = 156) over the control (CK = 75). The Stage 2 total was 389 cuttings. This cohort was balanced overall across the predefined DBH groups, comprising 198 Small DBH and 191 Large DBH cuttings, thereby maintaining comparable representation of the two groups in the survival analyses. Across both experimental stages, a total of 641 individual cuttings were monitored.

Following auxin treatment, cuttings were immediately planted to a depth of approximately 2 cm into their assigned substrates using transparent, flexible polyethylene pots (**Supplementary Figure 1C**). Transparent pots were selected specifically to enable non-destructive monitoring of root development. Adventitious root emergence and elongation at the basal cut surface and along the lower stem could be observed directly through the pot wall at each inspection without displacing or disturbing the cutting (**Supplementary Figure 1D**). Pots from different treatment combinations were distributed in a completely randomized spatial arrangement within the propagation chamber to prevent positional bias. Throughout the full observation period of both stages, with no reduction during a hardening-off phase, the propagation environment was held constant by automated misting once per day at a relative humidity of approximately 90%, approximating the humid microclimate characteristic of the Dawu Reserve where the donor population is established (Yang, 2007; Forestry Management Office, Pingtung Forest District, 2009; Ho et al., 2012). Air temperature was kept below 28 °C, and all cuttings received diffuse light through 70% shade netting, consistent with the shaded understorey conditions of the native habitat of *A. formosana*.

### 2.2 Data collection and definition of events

Both experiments were structured as time-to-event studies with the individual cutting serving as the primary analytical unit. We adopted cutting mortality rather than final adventitious rooting percentage as the primary event of interest because early rhizogenesis and post-rooting establishment are physiologically decoupled in *A. formosana*. Relying solely on a single terminal evaluation of rooting success can lead to a misleadingly optimistic assessment of propagation viability, as it fails to account for the high mortality risk that persists even after rhizogenesis. Therefore, our survival models specifically estimate the hazard of mortality.

The criterion for mortality was defined solely by aerial tissue symptoms, which included irreversible desiccation or mechanical collapse of the shoot tissue and complete abscission or total necrosis of all needle leaves. Inspections to assess these mortality criteria were carried out visually, without disturbing the cuttings, at a series of pre-scheduled dates spaced at irregular intervals (on average about one month apart, with the full schedule given below). When a cutting was confirmed dead based on these aerial symptoms, it was removed from the substrate. Mortality was scored as a single aerial-death endpoint (irreversible desiccation or necrosis of the shoot and needles), and losses were not partitioned by proximate cause, so fungal disease, basal rot, and mechanical breakage were not recorded as separate categories. Fungal infection in particular was controlled rather than tracked as an outcome: donors were pre-screened so that only healthy individuals were propagated (Section 2.1), and the cuttings received a routine prophylactic fungicide spray at approximately monthly intervals throughout the propagation period to suppress occasional flare-ups of latent parasitic fungi. Notably, post-mortem inspection of the basal cuts revealed that many cuttings had died despite having successfully initiated macroscopic roots (**Supplementary Figures 1E, F**). This observation confirmed the aforementioned physiological decoupling.

All cuttings were assessed at the pre-scheduled inspection dates throughout the observation periods. Consequently, the exact date of death was unknown for cuttings that died between two consecutive inspections. For operational analysis, event time was assigned as the number of days from cutting insertion to the inspection date at which death was first confirmed. Thus, the recorded event time represents the upper boundary of the interval during which death occurred. Because the same inspection dates applied to every cutting, the interval boundaries are common to the whole experiment. Stage 1 used 14 inspections, at days 30, 54, 92, 126, 191, 237, 298, 330, 353, 383, 413, 448, 494 and 516 after insertion. Stage 2 used 11, at days 58, 80, 108, 156, 181, 211, 241, 271, 315, 352 and 374. Interval widths ranged from 22 to 65 days in Stage 1, median 33, and from 22 to 58 days in Stage 2, median 30. Cuttings that remained alive at the final inspection were right-censored at day 516 in Stage 1 or day 374 in Stage 2. Monitoring and censoring were applied regardless of whether macroscopic roots had previously emerged. By the conclusion of the study, all surviving individuals had successfully initiated macroscopic root systems. Final survival therefore indicated not only successful macroscopic root initiation but also short-term post-rooting persistence under nursery conditions. The total observation periods spanned 516 days for Stage 1 and 374 days for Stage 2 to accommodate the characteristically protracted rooting and establishment dynamics of *A. formosana* under nursery conditions.

### 2.3 Statistical analysis

#### 2.3.1 Analytical framework and software

Because cutting mortality in *A. formosana* unfolds over a multi-month pre-rooting period, we combined endpoint analysis, kept for comparability with the prior Taxaceae propagation literature, with Kaplan-Meier estimation and Cox proportional hazards regression, which resolve the temporal structure of mortality that endpoint methods cannot access, following the approaches of Duarte et al. (2023) and Thomas et al. (2022). The fuller rationale for this two-level framework is given in **Supplementary Methods S1.3**. All analyses were implemented in Python 3.11. ANOVA was conducted using the ordinary least squares routine in statsmodels (v0.14), with *post-hoc* pairwise comparisons performed using Tukey’s honestly significant difference test where ANOVA indicated significant effects (α = 0.05). All survival analyses were performed using the lifelines library (v0.30.3) (Davidson-Pilon, 2019), specifically its KaplanMeierFitter, NelsonAalenFitter, and CoxPHFitter classes.

#### 2.3.2 Endpoint survival analysis

Endpoint survival was analyzed by ordinary least squares with Type II sums of squares in both stages. In Stage 1 the outcome was final survival coded as a binary variable (1 = alive, 0 = dead) at the final observation day, so that fit is a linear probability model (LPM). Although binary outcomes do not strictly satisfy the normality assumption of OLS, the LPM provides unbiased and consistent estimates of average marginal effects and avoids the complete separation problem that precludes maximum likelihood estimation in logistic regression when cell survival proportions reach 0% or 100%, as occurred in several substrate-by-PGR combinations in this study (Angrist and Pischke, 2009). The model included PGR treatment (CK, ABT, IAA, IBA, NAA), substrate (Substrate A through E), and their two-way interaction as fixed effects at the individual cutting level (n = 252). Because the PGR-by-substrate interaction was statistically significant, Tukey HSD *post-hoc* pairwise comparisons were conducted across all 25 PGR-by-substrate combinations to identify which specific combinations differed in final survival rate. In Stage 2, sample sizes were unequal across genotype-by-treatment cells. Survival rates were therefore first aggregated to the genotype × DBH group × PGR level to yield one mean survival rate per cell (n = 51 effective cells). The model was then fitted to these cell-level rates with DBH group (Small, Large), PGR treatment (CK, IBA, NAA), and their interaction as fixed effects. Because no significant effects were detected in Stage 2, no *post-hoc* comparisons were conducted. The Stage 2 endpoint model was used only as a comparative summary against conventional endpoint-based propagation assessments. Primary inference for Stage 2 was based on individual-level Cox proportional hazards modelling.

#### 2.3.3 Kaplan-Meier estimation, log-rank testing, and hazard rate estimation

Kaplan-Meier survival functions were estimated separately for each treatment group, DBH class, and PGR-by-DBH combination using the inspection date at which death was first confirmed as the operational event time. Because deaths could occur at any time between consecutive inspections, the resulting survival functions should be interpreted on the scale of the scheduled observation dates rather than as estimates of exact biological death times. For Stage 1, survival probabilities were tabulated at days 92, 191, and 360 as three reference time points spanning the 516-day observation window. For Stage 2, Kaplan-Meier survival curves were plotted across the full 374-day observation period. Furthermore, to systematically assess inter-genotypic survival variability, curves were generated for all 12 propagated genotypes. To clearly define the absolute boundaries of this genetic variation without the visual clutter of overlapping trajectories, a subset of four extreme genotypes (DG-15-1, DG-15-4, DG-15-6, and DG-15-9) was purposefully extracted for a focused panel. These four lineages were selected *post hoc* based on preliminary screening, as they exhibited either the most resilient or the most susceptible baseline survival profiles, effectively representing the maximal phenotypic spectrum of cutting viability.

Pairwise differences between survival curves were evaluated using the log-rank test (Mantel, 1966), which weights all event times equally. For Stage 2,log-rank tests were applied to assess survival differences associated with maternal DBH group, basal diameter group, plant height group, and donor sex. To characterize inter-genotypic survival variability, a multivariate log-rank test was applied across all 12 management units. All 66 pairwise genotype combinations were additionally compared using individual log-rank tests. Complete results are provided in **Supplementary Table 6**. The Nelson-Aalen estimator was additionally applied to each substrate-by-PGR combination toestimate and visualize the smoothed hazard rate h(t) stratified by rooting medium, using a kernelbandwidth of 40 days (Davidson-Pilon, 2019). This resolved discrete temporal peaks in cutting mortality risk. Pointwise 95% confidence bands for every substrate-by-PGR cell are given in **Supplementary Figure 5**.

#### 2.3.4 Cox proportional hazards modelling

Cox proportional hazards models were fitted to the individual-level time-to-event records from each stage to evaluate the simultaneous and interactive associations of experimental covariates with cutting mortality hazard (Thomas et al., 2022). In both models, cutting mortality was coded as the event indicator (1 = dead; 0 = right-censored). For dead cuttings, time was defined as the number of days from insertion to the inspection date at which death was first confirmed, so that the true death time is known only to lie in the half-open interval between the preceding inspection and that date. Surviving cuttings were censored at the final inspection date. In the Stage 1 model, covariates included substrate (reference: Substrate A), PGR treatment (reference: ABT), and their two-way interaction. ABT was designated as the PGR reference category because it represented a composite formulation distinct from the single-compound auxin treatments, thereby allowing direct comparison of each pure auxin against this composite baseline. In the Stage 2 model, covariates included continuous maternal DBH, PGR treatment (reference: CK), donor genotype, and two-way interaction terms for DBH × PGR and genotype × PGR.

Because deaths were detected only at these shared inspection dates, the observations have thestructure of a grouped-time survival design (Prentice and Gloeckler,1978). The Cox partial likelihood depends only on the rank order of the event times, so replacing each recorded time by any strictly increasing relabelling of a shared grid leaves every risk set, and therefore every coefficient, unchanged. Refitting on interval midpoints confirmed this (**Supplementary Table 8**). As a check that does not rest on the Cox structure, both models were also refitted asWeibull accelerated failure time models under the exact interval likelihood, in which no event timeis imputed (**Supplementary Table 9**).

The Stage 2 model includes genotype × PGR interaction terms, so stable estimation requires enough cuttings per genotype to fill each treatment cell. The model was therefore restricted to the five genotypes with the largest total samples, because genotypes with fewer cuttings produced treatment cells too sparse for stable interaction estimates, several of them with complete mortality. This criterion retained five genotypes (DG-15-6, DG-15-11, DG-15-12, DG-15-13, and DG-15-15), with DG-15–11 as the reference category and the remaining four entered as covariates. The remaining seven genotypes with sparse per-treatment data (DG-15-1, DG-15-2, DG-15-3, DG-15-4, DG-15-7, DG-15-9, and DG-15-14) were excluded from this specific modelling stage. These excluded genotypes were nonetheless retained in the Kaplan-Meier analyses, where their survival dynamics are described without parametric assumptions.

**Figure 1:**
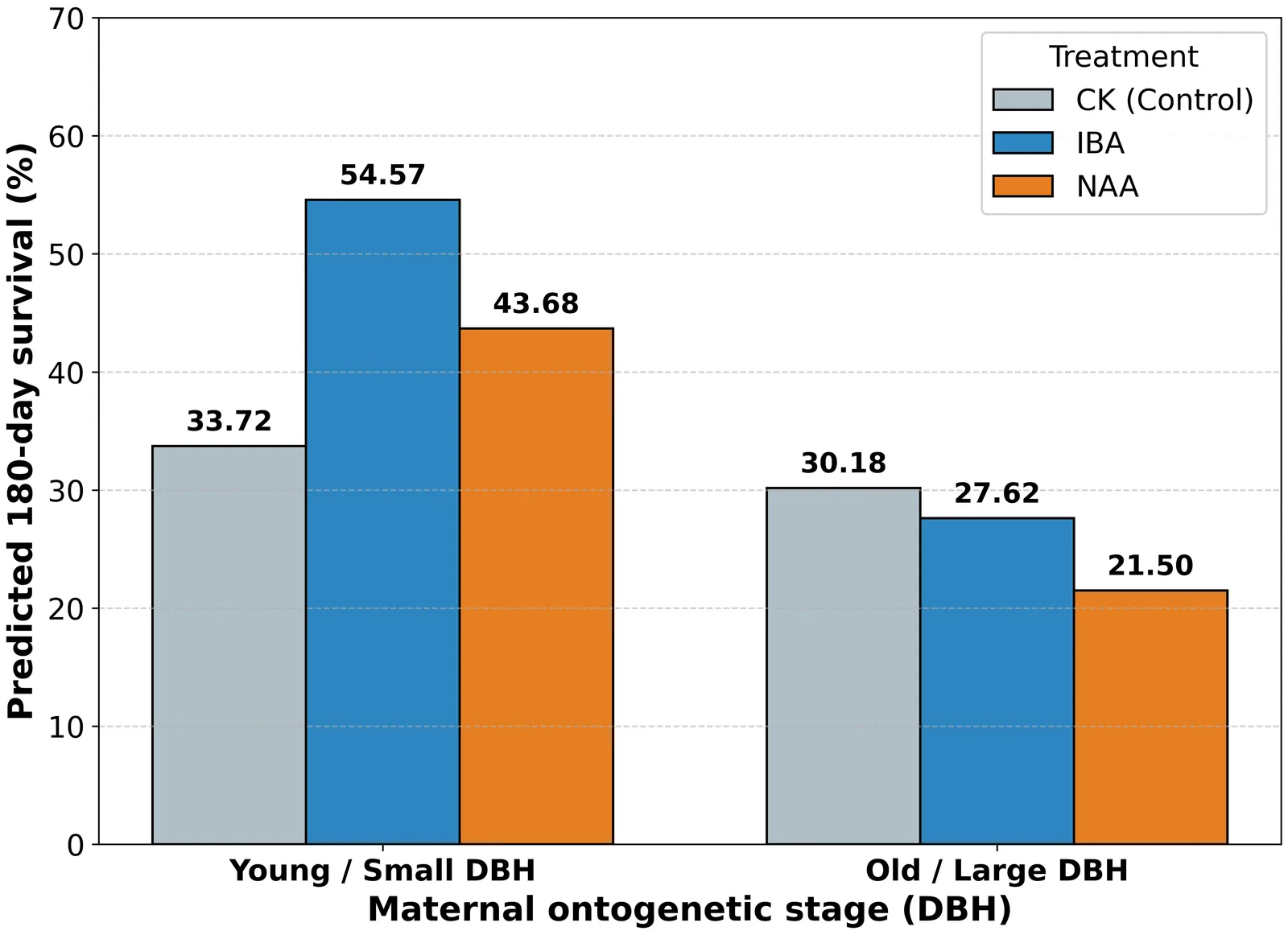
Model-predicted 180-day survival probabilities of *A. formosana* cuttings stratified by maternal ontogenetic stage (represented by DBH) and exogenous hormone treatments. Probabilities were derived from the Stage 2 multivariable Cox proportional hazards model of **Table 4** at a fixed covariate profile, with maternal DBH evaluated one standard deviation below and one standard deviation above the mean of the Cox analysis subset, at 2.79 cm and 15.25 cm, and genotype held at the reference unit DG-15-11. The bars labelled Small DBH and Large DBH therefore denote these two model profiles, not the donor groups formed by the median split in **Figure 2**, and the two figures are not directly comparable. The underlying event times are the scheduled inspection dates at which death was first confirmed.

**Figure 2:**
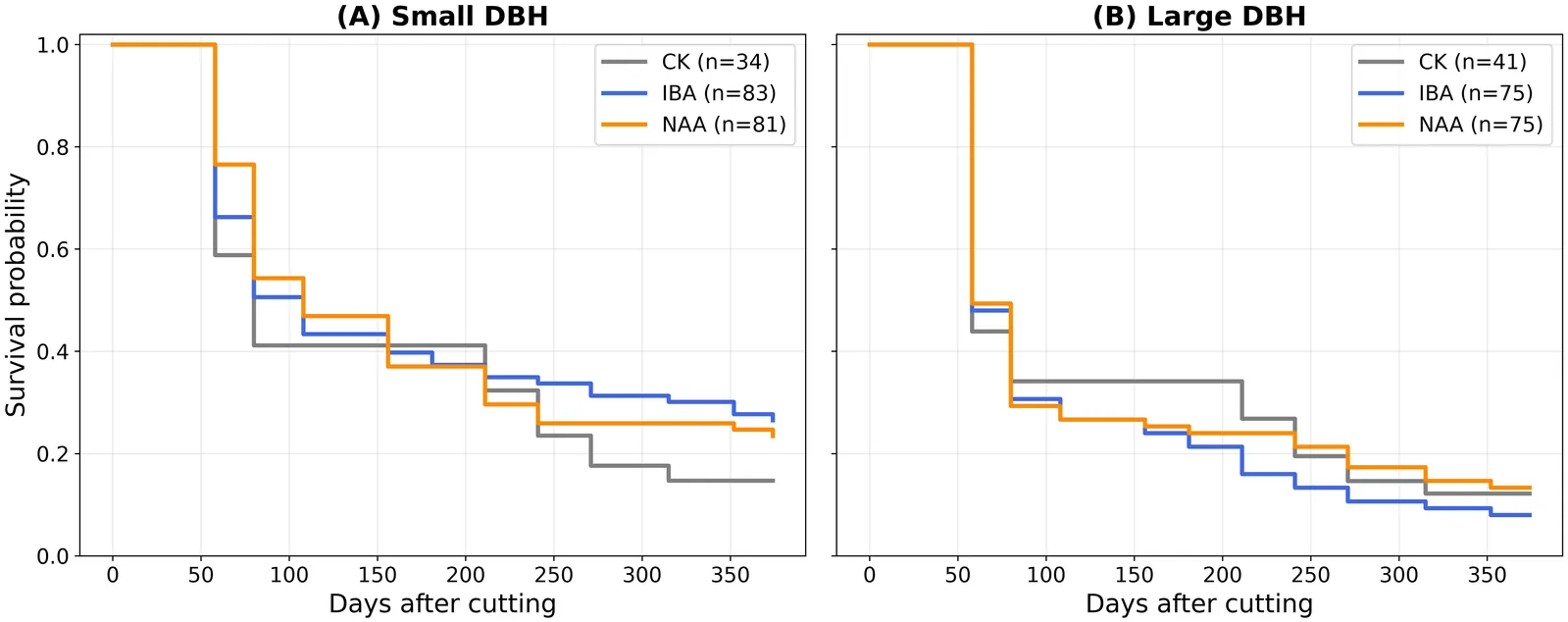
Kaplan-Meier survival curves of *A. formosana* cuttings in Stage 2, stratified by maternal ontogenetic stage (DBH group) and exogenous hormone treatment. Donor plants were classified into Small DBH (DBH < 7.5 cm) and Large DBH (DBH ≥ 7.5 cm) groups at the median DBH of the 33 donor trees. Panel **(A)** displays survival probabilities for cuttings derived from Small DBH donors and panel **(B)** for Large DBH donors. Each panel shows survival probabilities under three treatment conditions: an untreated control (CK), IBA, and NAA, pooled across all 12 management units. Event time is the scheduled inspection date at which death was first confirmed, so each curve steps at inspection dates rather than at exact times of death.

The DBH × PGR interaction was the term of primary biological interest because it testedwhether the association between maternal DBH and mortality hazard differed among PGR treatments, as hypothesised in the Introduction. A ridge penaliser (penaliser = 0.1) was applied to stabilise coefficient estimates given the number of parameters relative to events (Davidson-Pilon, 2019). In Stage 1 the penalty is needed for identification rather than for presentation. Three substrate-by-PGR cells lost every cutting, Substrate B with ABT at 8 of 8, Substrate D with ABT at 15 of 15, and Substrate E with CK at 7 of 7, and a fourth cell, Substrate A with IBA, lost only 1 of 8 against a reference cell that lost 7 of 8. With cells of that kind the unpenalized partial likelihood has no finite maximiser for the affected interaction contrasts, and the unpenalized fit does drive one coefficient to 4.385, a hazard ratio above 80, with a standard error of 1.346. The Stage 2 model carries no such degeneracy and remains estimable without a penalty, so there the ridge acts only as variance control. Both models were refitted across penaliser values of 0, 0.01, 0.05, 0.1, 0.2, 0.5 and 1, and the full profiles are reported in **Supplementary Table 11**. The Stage 1 model estimated 24 parameters from 169 mortality events (about 7.04 events per parameter), and the Stage 2 model estimated 17 parameters from 173 events among the 201 cuttings of the five retained genotypes (about 10.18 events per parameter). Stage 2 met the ten-events-per-parameter guideline, and although Stage 1 fell somewhat below it, the ridge penalty was applied specifically to stabilise the more heavily parameterised interaction terms. Hazard ratios with 95% Wald confidence intervals and corresponding p-values were extracted from each fitted model and presented as forest plots. Model-predicted survival probabilities at day 180 were additionally derived from this fitted Stage 2 model. These are predictions at a fixed covariate profile, not observed survival within a group of cuttings. Maternal DBH was evaluated one standard deviation below and one standard deviation above the mean of the Cox analysis subset, at 2.79 cm and 15.25 cm, with genotype held at the reference unit DG-15-11. The two profiles are labelled Small DBH and Large DBH in **Figure 1** to keep the terminology consistent across the paper, but they are not the two groups formed by the median split in **Figure 2**, so the values in the two figures are not interchangeable.

The proportional hazards assumption was assessed for each model using scaled Schoenfeld residualtests via the proportional_hazard_test function in lifelines (Davidson-Pilon, 2019), with full test statistics for all covariates provided in **Supplementary Table 7**. In Stage 1 none of the 24 terms departed from proportionality at α = 0.05. In Stage 2, 1 of the 17 terms did, against 0.9 expected by chance alone at that threshold. The assumption was satisfied for all primary covariates of biological interest in the Stage 2 model. A marginal violation was detected for one genotype main effect, suggesting that its baseline hazard relative to the reference category may not be strictly constant over time. This covariate was retained as a necessary confounder given that all of its interaction terms satisfied the proportional hazards assumption, and this limitation is acknowledged in the interpretation of genotype-specific results. A significance threshold of α = 0.05 was applied throughout all tests.

The stability of the Stage 2 estimates was checked in two ways. Sparse cells were tabulated,since 3 of the 15 genotype-by-treatment cells lost every cutting. The model was refitted on 500nonparametric bootstrap resamples, all of which converged, and the resampling distributions of the two reported interactions appear in **Supplementary Table 10**. Each resample refits the same penalized estimator, so these intervals describe reproducibility under resampling rather than unpenalized confidence intervals.

## 3 Results

### 3.1 Endpoint survival analysis: substrate dominance in Stage 1 and absence of detectable effects in Stage 2

Substrate had a stronger influence on final survival than PGR treatment. In Stage 1, a linearprobability model (Angrist and Pischke, 2009) showed asignificant substrate main effect (*p* = 0.004) and a significant PGR-by-substrate interaction (*p* = 0.008). The PGR main effect alone was not significant (*p* = 0.215). The significant interaction showed that the survival effect of each auxin treatment depended on the rooting medium (**Supplementary Table 2**). Tukey HSD comparisons identified five significant pairwise differences (**Supplementary Table 3**). IBA with peat-perlite gave the highest final survival (87.50 ± 12.50%). It exceeded ABT on shale-akadama mix, ABT on pure shredded shale, and CK on rockwool (all 0.00 ± 0.00%, *p* = 0.023, 0.003, and 0.036) and NAA on rockwool (9.09 ± 9.09%, *p* = 0.037). ABT on coconut-fibre mix reached 75.00 ± 16.37%, above ABT on pure shredded shale (*p* = 0.030). Zero-survival outcomes occurred under the ABT treatment in the shale-akadama mix and pure shredded shale, whereas final survival reached 75.00% in the coconut-fibre mix. This contrast indicates that the survival outcome associated with the ABT treatment was strongly substrate-dependent.

In Stage 2, no fixed effect was significant. The maternal DBH group(*p* = 0.069), PGR treatment (*p* = 0.328), andtheir interaction (*p* = 0.700) were all non-significant (**Supplementary Table 2**). The DBH group effect approached significance (*p* = 0.069). The time-to-event models resolved its basis (Sections 3.4 and 3.5).

### 3.2 Temporal dynamics of cutting mortality in Stage 1: substrate-stratified hazard profiles

**Figure 3:**
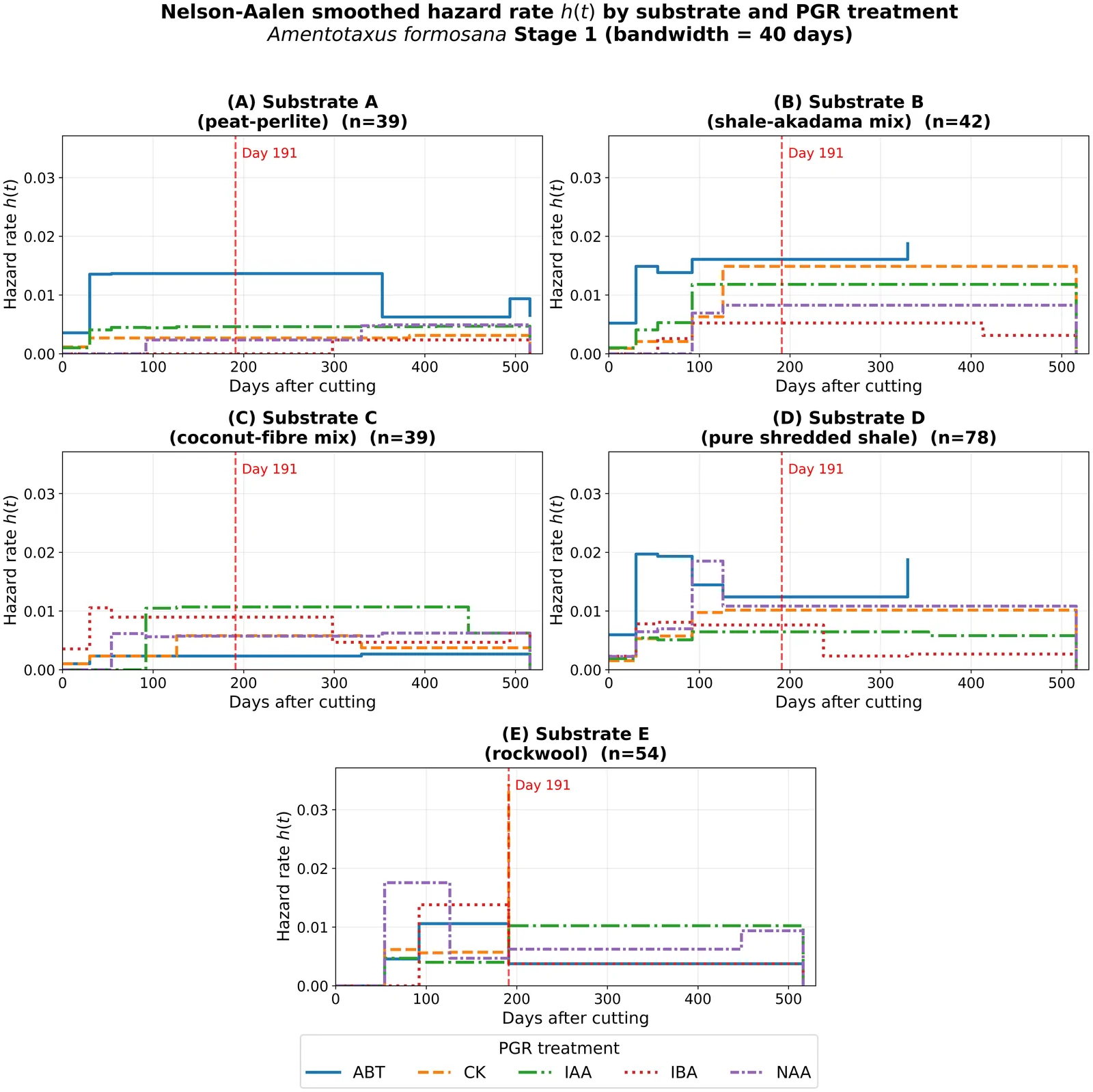
Substrate-stratified Nelson-Aalen smoothed instantaneous hazard rate h(t) of *A.formosana* cuttings under five PGR treatments in Stage 1 (kernel bandwidth = 40 days).**(A–E)** correspond to Substrate A through E, respectively, and each panel title gives the descriptive substrate name together with the number of cuttings contributing to that panel. All five panels are drawn on a common vertical scale, so hazard magnitudes can be compared directly across substrates. The red dashed vertical line marks day 191, the middle of the three reference time points used for the tabulated survival comparisons. Pointwise 95% confidence bands for each substrate-by-PGR cell are given in **Supplementary Figure 5**. Deaths were recorded only at the scheduled inspection dates, so the estimated hazard describes mortality risk on that inspection grid rather than at exact times of death.

Nelson-Aalen hazard profiles differed among the five substrates throughout Stage 1 (**Figure 3**). Cumulative mortality by day 191 ranged from 28.21% (peat-perlite) to 72.22% (rockwool),and by day 360 from 38.46% (peat-perlite) to 76.92% (pure shredded shale) (**Supplementary Table 4**). The substrates differed in the timing of mortality, not only in its final level.

Pure shredded shale had the highest and earliest mortality hazard (**Figure 3D**). Under most PGR treatments, h(t) peaked between days 30 and 92 and then declined. The exception was ABT, which rose again after day 300. By day 92, 64.10% of these cuttings had died, and Kaplan-Meier survival fell to 23.08% by day 360 (**Table 2**, **Supplementary Table 4**). Rockwool showed the opposite pattern (**Figure 3E**). Its hazard stayed near zero until about day 54, then rose to a peak between days 54 and 191, most under ABT and NAA. Rockwool mortality was delayed into the mid-observation period rather than early.

**Table 2:** Kaplan-Meier survival probability estimates of *A. formosana* cuttings across main independent factors and optimal combinations during stage 1 experiment.

Category	Factor/Combination	Day 92 (%)	Day 191 (%)	Day 360 (%)
Main Effect: Substrate	Substrate A	74.36	71.79	61.54
	Substrate B	54.76	40.48	38.10
	Substrate C	69.23	56.41	46.15
	Substrate D	35.90	29.49	23.08
	Substrate E	50.00	27.78	27.78
Main Effect: PGR	IBA	55.36	53.57	46.43
	NAA	52.17	39.13	32.61
	CK	66.67	39.58	37.50
	IAA	55.77	42.31	38.46
	ABT	36.00	32.00	24.00
Optimal Combinations (Survival ≥50% at Day 360)	Substrate A + CK	85.71	85.71	85.71
	Substrate A + IAA	62.50	50.00	50.00
	Substrate A + IBA	100.00	100.00	87.50
	Substrate A + NAA	87.50	87.50	62.50
	Substrate B + IBA	66.67	66.67	66.67
	Substrate B + NAA	75.00	50.00	50.00
	Substrate C + ABT	87.50	87.50	75.00
	Substrate C + CK	87.50	62.50	50.00

Substrate codes are A (peat-perlite), B (shale-akadama mix), C (coconut-fibre mix), D (pure shredded shale), and E (rockwool). Event time is the scheduled inspection date at which death was first confirmed. Day 360 is a reference readout point and not an inspection date. The Stage 1 inspections fell on days 30, 54, 92, 126, 191, 237, 298, 330, 353, 383, 413, 448, 494 and 516, so the day-360 column carries the estimate from the last inspection on or before it, on day 353.

Peat-perlite had the lowest and most even hazard of all substrates (**Figure 3A**). The exception was again ABT, which produced an early peak within the first 30 days.Peat-perlite kept a low but non-zero h(t) across the full 516-day window. Cumulative mortalityaccumulated most gradually of all five substrates (25.64%, 28.21%, and 38.46% at days 92, 191, and 360, **Supplementary Table 4**). It held the highest Kaplan-Meier survival throughout (74.36%, 71.79%, and 61.54% at days 92, 191, and 360, **Table 2**). This was ahead of the next-best substrate, coconut-fibre mix (69.23%, 56.41%, and 46.15%, respectively). The two intermediate substrates differed in shape. Shale-akadama mix had a moderate early peak that subsided after day 92 but plateaued through day 191 under several treatments (**Figure 3B**). Coconut-fibre mix was biphasic, with a second risk window near days 92 to 191 that peat-perlite lacked (**Figure 3C**).

Optimal substrate-PGR combinations were defined as those maintaining at least 50% Kaplan-Meier survival at day 360. Eight of the 25 combinations met this criterion (**Table 2**). The highest was peat-perlite + IBA (100.00%, 100.00%, and 87.50% at days 92, 191, and 360), with no mortality in the first 191 days and cuttings that kept well-developed roots and stayed viable beyond 13 months (**Supplementary Figure 1G**). Peat-perlite + CK (85.71% at day 360) and peat-perlite + NAA (62.50%) also held high long-term survival. Peat-perlite + IAA met the threshold exactly (50.00%). Four combinations on higher-mortality substrates also passed it, coconut-fibre mix + ABT (75.00%), shale-akadama mix + IBA (66.67%), shale-akadama mix + NAA (50.00%), and coconut-fibre mix + CK (50.00%).

### 3.3 Multivariable Cox proportional hazards analysis of stage 1: substrate and PGR effects on mortality hazard

The Stage 1 Cox model identified significant substrate and PGR coefficients, as well as one significant substrate × PGR interaction (**Table 3**, peat-perlite and ABT as reference categories). Among substrate main effects, pure shredded shale was the only rooting medium to significantly elevate the hazard (HR = 2.338, 95% CI: 1.405–3.891, *p* = 0.001). Shale-akadama mix (HR = 1.837, 95% CI: 0.970–3.479, *p* = 0.062), coconut-fibre mix (HR = 0.658, 95% CI: 0.349–1.242, *p* = 0.197), and rockwool (HR = 1.070, 95% CI: 0.617–1.855, *p* = 0.811) did not differ significantly from peat-perlite. The non-significant HR below 1.000 for coconut-fibre mix was nonetheless consistent with its intermediate day-360 Kaplan-Meier survival (46.15%, **Table 2**). This indicated that its survival deficit relative to peat-perlite reflected an endpoint-level difference rather than a systematically higher mortality rate.

**Table 3:** Full multivariable cox pHazards model assessing the main and interaction effects of substrate and hormone treatments on cutting mortality (stage 1 experiment).

Covariate	Coefficient (*β*)	SE	Hazard ratio (HR)	95% Confidence interval (CI)	*p*-value
Main effect: substrate (Ref: Substrate A)					
Substrate B	0.608	0.326	1.837	0.970 – 3.479	0.062
Substrate C	-0.418	0.324	0.658	0.349 – 1.242	0.197
Substrate D	0.849	0.260	2.338	1.405 – 3.891	0.001**
Substrate E	0.067	0.281	1.070	0.617 – 1.855	0.811
Main effect: PGR (Ref: ABT)					
CK (Control)	-0.505	0.314	0.604	0.326 – 1.116	0.107
IAA	-0.205	0.301	0.814	0.452 – 1.468	0.495
IBA	-0.659	0.297	0.517	0.289 – 0.926	0.027*
NAA	-0.415	0.306	0.660	0.362 – 1.203	0.175
Interaction effect: substrate × PGR					
Substrate B × CK	-0.222	0.521	0.801	0.289 – 2.223	0.670
Substrate C × CK	0.278	0.578	1.320	0.425 – 4.102	0.631
Substrate D × CK	-0.199	0.414	0.819	0.364 – 1.845	0.630
Substrate E × CK	0.851	0.509	2.341	0.863 – 6.350	0.095
Substrate B × IAA	-0.395	0.537	0.674	0.235 – 1.929	0.462
Substrate C × IAA	0.500	0.529	1.648	0.584 – 4.651	0.345
Substrate D × IAA	-0.645	0.429	0.525	0.226 – 1.217	0.133
Substrate E × IAA	-0.053	0.442	0.948	0.398 – 2.256	0.904
Substrate B × IBA	-0.377	0.529	0.686	0.243 – 1.934	0.476
Substrate C × IBA	1.120	0.538	3.066	1.069 – 8.793	0.037*
Substrate D × IBA	-0.028	0.393	0.973	0.450 – 2.100	0.943
Substrate E × IBA	0.354	0.478	1.424	0.558 – 3.633	0.459
Substrate B × NAA	-0.648	0.556	0.523	0.176 – 1.554	0.243
Substrate C × NAA	0.716	0.562	2.046	0.681 – 6.153	0.202
Substrate D × NAA	0.082	0.437	1.086	0.461 – 2.556	0.850
Substrate E × NAA	0.844	0.455	2.325	0.953 – 5.672	0.064

Hazard Ratio < 1 indicates a reduced mortality risk, while HR > 1 indicates an increasedrisk. Significance codes: ** *p* < 0.01, * *p*< 0.05. Interaction terms are presented for Substrate B through E only. Substrate A interactions are subsumed into the PGR main effects, which represent PGR-specific hazard ratios within the Substrate A reference substrate. Substrate codes are A (peat-perlite), B (shale-akadama mix), C (coconut-fibre mix), D (pure shredded shale), and E (rockwool). The model carries a ridge penalty of 0.1, and **Supplementary Table 11** gives the hazard ratios across penaliser values from 0 to 1. Event time is the scheduled inspection date at which death was first confirmed.

Among PGR treatments, only IBA reduced the hazard significantly relative to the ABT composite, to about half (HR = 0.517, 95% CI: 0.289–0.926, *p* = 0.027). This matched its highest day-360 survival among treatments (46.43%, **Table 2**). CK, IAA, and NAA did not differ significantly from ABT (all *p* > 0.10, **Table 3**). All three still had hazard ratios below 1.000, so ABT carried the highest hazard of thefive treatments, matching its zero final survival in shale-akadama mix and pure shredded shale(**Supplementary Table 3**).

Of the 16 substrate-by-PGR interaction terms, only coconut-fibre mix × IBA was significant (HR = 3.066, 95% CI: 1.069–8.793, *p* = 0.037, **Table 3**). The IBA main effect represented the IBA-versus-ABT contrast within the peat-perlite reference substrate. The positive coconut-fibre mix × IBA interaction therefore indicated that this contrast was substantially attenuated, and potentially reversed, in the coconut-fibre mix. The Kaplan-Meier estimates showed the same contrast. Peat-perlite with IBA reached 87.50% survival at day 360 (**Table 2**), whereas coconut-fibre mix with IBA reached only 37.50% (**Supplementary Table 5**). The other 15 interaction terms were all non-significant (**Table 3**).

### 3.4 Kaplan-Meier survival analysis of stage 2: effects of maternal ontogeny, morphological traits, donor sex, and genotype on cutting survival

The maternal traits were strongly intercorrelated across the 33 donor trees (**Supplementary Figure 2**). DBH and BD were nearly collinear (*r* = 0.982), and DBH and plantheight were strongly correlated (*r* = 0.891). The DBH and BD medians split the trees identically, so their log-rank results were identical (χ² = 15.418, *p* < 0.001). Plant height gave the same pattern, with taller donors showing lower survival (χ² = 15.133, *p* < 0.001, **Supplementary Figure 3B**). We therefore kept DBH as the only continuous morphological predictor in the Stage 2 Cox model to avoid redundant parameters.

Kaplan-Meier survival curves stratified by maternal DBH group and PGR treatment are presented in **Figure 2**. Cuttings from Small DBH donors maintained higher survival than those from Large DBH donors under all three PGR conditions across the 374-day period (**Figure 2**). Donors were split at the median DBH of 7.5 cm into Small (< 7.5 cm) and Large (≥ 7.5 cm) groups, pooled across the 12 management units. Under CK the two groups ran together until about day 180, after which Large DBH cuttings declined more steeply. Under IBA the split began earlier and was larger, with Large DBH mortality concentrated over the first three inspections, from day 58 to day 108 (**Figures 2A, B**). The DBH-group difference was significant (*p* < 0.001, **Supplementary Figure 3A**).

Donor sex could be assigned for only 6 of the 33 trees, because the rest were juvenile or non-reproductive during the survey (**Supplementary Table 1**). Survival did not differ between the sexes (χ² = 0.026,*p* = 0.873, **Supplementary Figure 3C**). The small sexed sample left this comparison underpowered.

The overall difference among the 12 genotypes was significant (χ² = 63.441,*p* < 0.001, **Supplementary Figure 4**). Stratified by treatment, the difference was not significant under CK (χ² = 11.904, *p* = 0.371) but was significant under IBA (χ² = 37.237, *p* < 0.001) and NAA (χ² = 52.192, *p* < 0.001). In treatment-stratified log-rank analyses, among-genotype survival differences were statistically detectable under IBA and NAA but not under CK.

Of the 66 genotype pairwise comparisons, 33 were statistically significant(*p* < 0.05, **Supplementary Table 6**). **Supplementary Figure 3D** illustrates the range of inter-genotypic variation, with four of the 12 genotypes selected*post hoc* to represent the survival extremes. DG-15–4 came entirely fromSmall DBH donors (mean 1.6 cm) and had the highest survival (S(374) = 0.400), significantly above eight other genotypes (DG-15-2, DG-15-6, DG-15-7, DG-15-9, DG-15-11, DG-15-12, DG-15-13, and DG-15-15, all *p* < 0.05) (**Supplementary Table 6**). DG-15–1 was represented by Large DBH donors (mean 18.0 cm) but stillmaintained relatively high survival (S(374) = 0.286). DG-15–9 and DG-15-6, also Large DBH(mean 25.8 and 10.6 cm), had the lowest survival (S(374) = 0.000 and 0.038) and fell significantly below nine and eight other genotypes (**Supplementary Table 6**). DG-15-1, DG-15-6, and DG-15–9 all belong to the Large DBH group, yet their survivaloutcomes diverged markedly. DG-15–9 and DG-15–6 did not differ significantly from eachother (*χ²* = 1.069, *p* = 0.301) (**Supplementary Table 6**). These descriptive contrasts suggest that donor size alone did not account for the observed among-genotype survival variation.

### 3.5 Cox proportional hazards modelling of stage 2: a maternal DBH × IBA interaction and a genotype-specific NAA response

**Figure 4:**
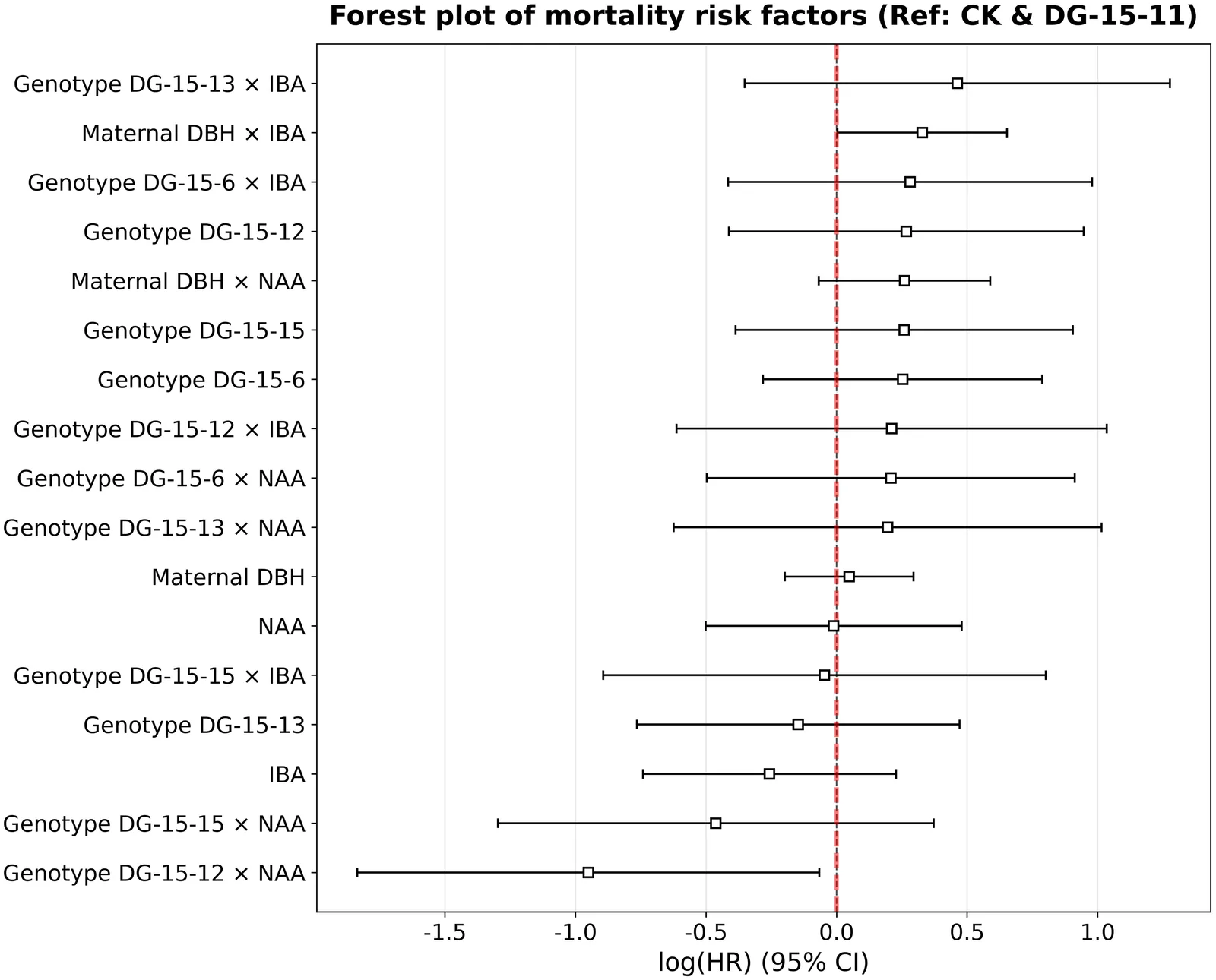
Estimated log-transformed hazard ratios (HR) for mortality risk factors in *A. formosana* cuttings. Positive and negative values on the x-axis denote increased and reduced risk compared to the reference categories of the Stage 2 model of **Table 4**, the untreated control (CK) for treatment and DG-15–11 for genotype. The three maternal DBH terms are slopes on a standardized continuous covariate, so each gives the change in hazard per one standard deviation of 6.23 cm rather than a contrast with a reference category. Horizontal error bars represent 95% confidence intervals (CI). Event time is the scheduled inspection date at which death was first confirmed.

The Stage 2 multivariable Cox proportional hazards model returned non-significant main effects for continuous maternal DBH (HR = 1.050, 95% CI: 0.820–1.343, *p* = 0.699), IBA (HR = 0.774, 95% CI: 0.476–1.256, *p* = 0.299), NAA (HR = 0.989, 95% CI: 0.606–1.615, *p* = 0.964), and all four genotype terms representing their contrasts with the DG-15–11 reference under CK (DG-15-6, DG-15-12, DG-15-13, and DG-15-15; all *p* > 0.05). The criteria used to retain genotypes in this model are described in Section 2.3.4. Estimated hazard ratios and 95% confidence intervals for all covariates and interaction terms are presented in **Table 4** and visualized in the forest plot in **Figure 4**.

**Table 4:** Multivariable cox proportional hazards model assessing the interaction effects of maternal DBH, genotype, and PGR treatments on cutting mortality (Stage 2 experiment).

Covariate	Coefficient (*β*)	SE	Hazard ratio (HR)	95% Confidence interval (CI)	*p*-value
Main effects					
DBH	0.049	0.126	1.050	0.820 – 1.343	0.699
IBA	-0.257	0.247	0.774	0.476 – 1.256	0.299
NAA	-0.011	0.250	0.989	0.606 – 1.615	0.964
Genotype DG-15-6	0.253	0.273	1.288	0.754 – 2.199	0.354
Genotype DG-15-12	0.267	0.347	1.306	0.662 – 2.578	0.441
Genotype DG-15-13	-0.147	0.315	0.864	0.466 – 1.602	0.642
Genotype DG-15-15	0.259	0.330	1.296	0.679 – 2.472	0.432
Interaction effects					
Maternal DBH × IBA	0.328	0.166	1.388	1.003 – 1.922	0.048*
Maternal DBH × NAA	0.260	0.168	1.298	0.934 – 1.802	0.120
Genotype DG-15-6 × IBA	0.282	0.356	1.326	0.660 – 2.662	0.428
Genotype DG-15-6 × NAA	0.208	0.360	1.231	0.609 – 2.491	0.563
Genotype DG-15-12 × IBA	0.211	0.421	1.235	0.542 – 2.816	0.616
Genotype DG-15-12 × NAA	-0.951	0.452	0.386	0.159 – 0.936	0.035*
Genotype DG-15-13 × IBA	0.463	0.416	1.588	0.703 – 3.588	0.266
Genotype DG-15-13 × NAA	0.196	0.418	1.216	0.536 – 2.759	0.640
Genotype DG-15-15 × IBA	-0.046	0.432	0.955	0.409 – 2.229	0.915
Genotype DG-15-15 × NAA	-0.462	0.426	0.630	0.273 – 1.451	0.277

Only the five major genotypes with sufficient sample sizes were included in this model. Baseline reference categories are control (CK) and genotype DG-15-11.

Hazard Ratio < 1 indicates a reduced mortality risk, while HR > 1 indicates an increasedrisk. Significance codes: *p < 0.05. DBH is standardized, so the DBHhazard ratio is the change per one standard deviation of 6.23 cm. The model carries a ridge penalty of 0.1, and **Supplementary Table 11** gives the hazard ratios across penaliser values from 0 to 1. Event time is the scheduled inspection date at which death was first confirmed.

The maternal DBH × IBA interaction was statistically significant (interaction HR = 1.388, 95% CI: 1.003–1.922, p = 0.048; **Table 4**, **Figure 4**), indicating that the association between maternal DBH and mortality hazard differed between IBA and the untreated control. The maternal DBH main effect, which represents the DBH-associated change in hazard under the CK reference treatment, was not significant (HR = 1.050, 95% CI: 0.820–1.343, p = 0.699). Thus, there was no statistical evidence of a DBH-associated change in mortality hazard under CK. By contrast, the positive DBH × IBA interaction indicated that the DBH-associated hazard slope was steeper under IBA than under CK. The maternal DBH × NAA interaction was also positive but did not reach statistical significance (interaction HR = 1.298, 95% CI: 0.934–1.802, p = 0.120; **Table 4**). Model-predicted survival probabilities at day 180 illustrated these treatment-dependent patterns (**Figure 1**). These are predictions at a fixed covariate profile, with maternal DBH set one standard deviation below and one standard deviation above the subset mean, at 2.79 cm and 15.25 cm, and genotype held at the reference unit DG-15-11 (Section 2.3.4). Predicted survival under IBA was 54.57% at the lower DBH profile and 27.62% at the upper, a difference of 26.95 percentage points. The corresponding predicted differences were 22.18 percentage points under NAA (43.68% vs. 21.50%) and 3.54 percentage points under CK (33.72% vs. 30.18%). These model predictions suggest that the survival advantage associated with IBA was more apparent among cuttings from smaller donors, whereas no comparable advantage was apparent among cuttings from larger donors. Because these values fix genotype and evaluate DBH at two points on a continuous scale, they are larger than the Kaplan-Meier differences between the median split groups shown in **Figure 2** and should not be read as observed group survival.

Among genotype × PGR interaction terms, Genotype DG-15-12 × NAA was the only significant interaction (interaction HR = 0.386, 95% CI: 0.159–0.936, *p* = 0.035; **Table 4**, **Figure 4**). This coefficient indicates that the NAA-associated change in hazard for DG-15–12differed significantly from the corresponding treatment contrast for the DG-15–11 referencegenotype. Consistent with this interaction, DG-15–12 had no surviving cuttings at the end of the CK or IBA treatments, whereas its final survival under NAA was 46.67% (**Supplementary Figure 4**). All other genotype-by-PGR interaction terms were non-significant (*p* > 0.05) (**Table 4**).

Scaled Schoenfeld residual tests checked whether each covariate’s effect on the hazardstayed constant over the study, the Cox model’s central assumption. It held for every primarycovariate (**Supplementary Table 7**): DBH (χ² = 0.008, *p* = 0.930), IBA (χ² =0.441, *p* = 0.507), NAA (χ² = 0.612,*p* = 0.434), Maternal DBH × IBA (χ² = 0.173, *p* = 0.678), Maternal DBH × NAA (χ² = 0.784, *p* = 0.376), and all genotype-by-PGR interaction terms. The one exception was the DG-15–6 main effect, which showed a marginal violation (rank-based test: χ² = 5.354, *p* = 0.021). Its hazard relative to the DG-15–11 reference therefore probably shifted over the observation period rather than holding steady. We kept the term because all of its interaction terms met the assumption, but interpreted its main-effect coefficient cautiously. The Stage 1 model was tested in the same way. None of its 24 terms departed from proportionality at α = 0.05 (**Supplementary Table 7**).

## 4 Discussion

The two-stage experimental framework and Cox proportional hazards modelling suggest that cutting mortality in *A. formosana* varies with substrate type, PGR treatment, maternal DBH, and genotype, with statistically detectable interactions in specific factor combinations.

### 4.1 Substrate-dependent mortality dynamics and the high mortality associated with the ABT treatment

Substrate composition had a significant main effect on cutting survival and interacted significantly with PGR treatment in Stage 1. The Cox model identified pure shredded shale as the sole substrate that significantly elevated the mortality hazard relative to peat-perlite. The Nelson-Aalen hazard profiles provided descriptive information on the timing of mortality and suggested possible substrate-related mechanisms. Pure shredded shale exhibited a sharp, early-concentrated mortality peak between days 30 and 92, by which time 64.10% of all cuttings had already died. This temporal pattern is consistent with, but does not directly demonstrate, rapid desiccation stress in a coarse, free-draining substrate with limited moisture-buffering capacity. Because substrate water content, air-filled porosity, and cutting water status were not measured, desiccation remains a working explanation for the early mortality peak (Puri and Verma, 1996; Druege, 2020). Despite the ecological rationale for incorporating shredded shale to emulate the stony, colluvial soils of the species’ native upper-slope habitat (Yang, 2007; Ho et al., 2012), the poor survival observed in pure shale indicates that directly reproducing this coarse lithic texture did not provide a favourable nursery substrate under the conditions tested.

Rockwool displayed a contrasting but equally informative pattern, with near-zero mortality through approximately day 54 followed by a concentrated mortality peak between days 54 and 191. This delayed-onset profile is consistent with initial protection from desiccation afforded by the continuously pre-moistened substrate, followed by a later increase in mortality that may reflect excessive water retention, declining substrate aeration, or other time-dependent stress processes (Xavier et al., 2021; Duarte et al., 2023). These alternatives cannot be distinguished because substrate oxygen status and water content were not measured. A comparable substrate-dependent mortality dynamic has been documented in *T. baccata*, where cuttings planted in pure river sand showed complete failure, while a forest soil, peat, and farmyard manure mixture (1:1:1) supported optimal rooting (Das and Jha, 2018a). Peat-perlite consistently maintained the lowest and most temporally diffuse hazard profile, sustaining the highest Kaplan-Meier survival across all three reference time points. The highest single-combination survival in the entire experiment was peat-perlite + IBA, a pattern consistent with the generally favourable balance between water retention and aeration reported for peat-perlite formulations. The functional correspondence between peat-perlite and the moist but well-drained colluvial soils where *A. formosana* naturally establishes (Yang, 2007; Ho et al., 2012) suggests that optimal propagation substrates need not replicate the lithic texture of native soils, but rather their hydrological function of sustained moisture with adequate aeration.

The ABT composite rooting powder, containing IAA and NAA in a fixed ratio (Wang, 1989), was associated with the highest mortality hazard among all five PGRtreatments, with all other treatments returning hazard ratios below 1.000 relative to the ABT reference. IBA was the only treatment that significantly reduced mortality risk relative to ABT (HR = 0.517, *p* = 0.027). However, this pattern was not uniform across substrates: ABT-treated cuttings achieved 75.00% final survival on coconut-fibre mix while producing zero or near-zero survival on shale-akadama mix, pure shredded shale, and rockwool. The fixed-ratio composition of ABT precludes adjustment to species-specific tolerance thresholds. The combined auxin dosage may have exceeded the detoxification capacity of *A. formosana* tissues under certain hydrological conditions. This is consistent with the extreme NAA sensitivity of the closely related *T. wallichiana*, where NAA application caused 100% mortality from basal necrosis (Das and Jha, 2014). It also fits the broader pattern across woody species, in which NAA carries a consistently higher phytotoxicity risk than IBA, a difference usually explained by its narrower margin between effective and toxic doses (Maynard and Johnson, 1993; Trueman and Richardson, 2008; Faganello et al., 2015; Druege, 2020). The notable exception of coconut-fibre mix may be attributed to the cation exchange capacity and physical adsorption properties of coconut fibre, which may reduce auxin bioavailability at the cutting base below the phytotoxicity threshold. If such sequestration occurs, it would not be selective for one auxin, and this reading is consistent with the significant coconut-fibre mix × IBA interaction (HR = 3.066, *p* = 0.037). That interaction rests on a single cell of eight cuttings and does not reach significance under the interval-censored Weibull refit (**Supplementary Table 9**), so it carries less weight than the substrate main effect. The protective effect of IBA observed in peat-perlite was substantially weakened in coconut fibre. The reciprocal pattern, in which survival under ABT was highest on coconut-fibre mix while the survival advantage of IBA was smallest there, is what a substrate acting on auxin availability in general rather than on one particular compound would be expected to produce. The present study did not directly measure tissue auxin concentrations or physiological stress markers, so these mechanistic interpretations remain working hypotheses consistent with the observed survival trajectories but not directly validated by physiological data.

### 4.2 Maternal DBH modifies the association between IBA and cutting mortality

The Stage 2 Cox model supported a treatment-dependent association between maternal DBH and cutting mortality. The maternal DBH × IBA interaction was statistically significant, indicating that the DBH-associated hazard slope differed between IBA and the untreated control. The interaction HR of 1.388 represents the ratio between these two DBH-associated slopes rather than the hazard increase within the IBA treatment alone. Consistent with this interaction, model-predicted survival at day 180 differed more strongly between the two DBH profiles of **Figure 1** under IBA than under CK. Predicted survival under IBA was 54.57% at the lower DBH profile and 27.62% at the upper, a difference of 26.95 percentage points, whereas the corresponding difference under CK was 3.54 percentage points (33.72% vs. 30.18%). These patterns suggest that the association between increasing maternal DBH and mortality was more pronounced under IBA than under the untreated control. The maternal DBH × NAA interaction was also positive but did not reach statistical significance, although the model-predicted survival difference between the two DBH profiles under NAA was 22.18 percentage points.

This pattern is consistent with the widely reported decline in rooting competence associated with donor maturation in Taxaceae and other woody species. Reduced propagation success from mature donors has been documented in related taxa such as *Taxus baccata* and in other woody species, including *Pinus radiata*, *Prunus avium*, and *Tilia mandshurica* (Dick and Leakey, 2006; Álvarez et al., 2016; Das and Jha, 2018b; Mu et al., 2022; Savev and Iliev, 2023). In these systems, donor maturation has been associated with altered endogenous hormone balance, increased lignification, reduced cellular competence for adventitious-root initiation, and prolonged dependence on stored carbohydrate reserves. Such changes could delay root formation and increase the period during which cuttings remain vulnerable to carbon depletion and mortality before functional roots are established (Costa, 2002; Abu-Abied et al., 2014; Aumond et al., 2017; Druege, 2020; Chang et al., 2023). Recent syntheses similarly identify auxin as a central regulator of adventitious-root induction acting within a broader hormone network that changes as donor tissues mature (Rasmussen et al., 2015; Adem et al., 2024; Liu et al., 2025). In the present study, however, endogenous hormone concentrations, carbohydrate reserves, lignification, and the developmental stages of root initiation were not measured. These mechanisms therefore provide biologically plausible explanations for the observed DBH-dependent treatment pattern, but they remain working hypotheses rather than directly tested causal pathways.

Beyond the ontogenetic gradient, descriptive analyses (Kaplan-Meier and log-rank) suggest that genotype identity contributes additional heterogeneity to cutting survival. We note that the strength of this evidence varies by analytical approach. Although descriptive Kaplan-Meier analyses across all twelve sampled genotypes revealed pronounced inter-genotypic differences (e.g., DG-15–4 and DG-15-9), several of these genotypes were excluded from the multivariable Cox model owing to insufficient per-treatment sample sizes. The genotype effects therefore rest on two complementary but unequal evidentiary layers, descriptive ranking and formal multivariable inference, a distinction we maintain throughout the following discussion. The pronounced survival divergence among genotypes within the Large DBH group further indicates that donor size alone does not account for cutting outcome. DG-15–1 maintained moderate descriptive survival, whereas DG-15–9 and DG-15-6, represented by donors of similarly large DBH, showed much lower survival. Maternal DBH and genotype identity therefore appear to represent partly independent sources of variation in cutting mortality. For *A. formosana*, this distinction is relevant to conservation propagation because the species commonly regenerates through basal sprouting, making physiological age difficult to infer from DBH or tree height alone. Propagation programmes should therefore consider both donor size and genotype identity when allocating limited shoot material, while recognising that the present genotype comparisons differ in sample size and level of statistical support.

### 4.3 Genotype × auxin interactions and implications for conservation propagation

Treatment-stratified log-rank analyses detected significant among-genotype survival differences under IBA and NAA but not under CK. This pattern suggests that genotypic variation in survival was more evident under the two auxin treatments, although the stratified tests do not themselves constitute a formal genotype × treatment interaction test. Of the 66 pairwise genotype comparisons, 33 reached significance. The significant DG-15-12 × NAA interaction indicates that the NAA-versus-CK treatment contrast for DG-15–12 differed significantly from the corresponding contrast for the DG-15–11 reference genotype, providing evidence that the association between auxin treatment and mortality can differ among genotypes. This finding is consistent with the principle established across diverse plant taxa that genotype-dependent auxin responses can be practically important in vegetative propagation (Martínez et al., 2020), as also demonstrated in *Eucalyptus* hybrids (Nakhooda et al., 2014) and *Triplochiton scleroxylon* (Leakey et al., 1982).

For *A. formosana*, these genotype-dependent treatment patterns have practical implications for the design of conservation propagation trials. While prior trials highlighted extreme inter-donor variability and severe late-stage mortality under uniform conditions (Chung et al., 2019), the present study extends these findings by identifying factors associated with this variation. Maternal DBH and genotype-specific treatment responses appear to contribute jointly to variation in cutting mortality. These factors can be incorporated into survival models to inform the design of subsequent propagation trials. Practically, the data support a three-tiered, provisional prioritization strategy that requires further validation in expanded experimental cohorts. First, the significant maternal DBH × IBA interaction indicates that a uniform high-concentration IBA protocol may not perform consistently across donor sizes. Cuttings from smaller-DBH donors may be prioritised for initial IBA trials, while alternative concentrations or exposure durations should be tested experimentally for larger-DBH donors. Second, based on pooled Kaplan-Meier survival rankings rather than multivariable inference, genotypes with high descriptive cutting viability such as DG-15–4 and DG-15–1 may merit initial prioritisation. This recommendation rests on descriptive evidence and should be treated as a working hypothesis pending confirmation in larger-sample studies to maximize seedling output with limited resources. Third, genotype-specific pilot testing of auxin treatments appears warranted. The observed survival pattern of DG-15–12 was more favourable under NAA than under CK or IBA, and small-scale pilot trials pairing individual genotypes with candidate auxin treatments are recommended before committing scarce wild-collected material to large-scale propagation. The 29 genetic conservation units established by Ko et al. (2025) across the three extant populations provide a framework for implementing such genotype-matched protocols, and this is particularly important given the extremely low within-population genetic diversity and high inter-population differentiation characteristic of *Amentotaxus* (Ge et al., 2005; Ko et al., 2025). As demonstrated in *T. yunnanensis*, where cultivated *ex situ* collections captured only a fraction of wild genetic diversity due to uneven propagation success among founders (Miao et al., 2015), the loss of any propagation-recalcitrant genotype may represent disproportionate loss of species-level allelic variation. The present study is, to our knowledge, the first systematic characterization of genotype × auxin interactions in any *Amentotaxus* species, expanding a propagation evidence base that previously comprised only the aforementioned endpoint study on *A. formosana* (Chung et al., 2019) and a brief report on *A. argotaenia* demonstrating approximately 60% axillary bud induction *in vitro* (Yu et al., 2005).

### 4.4 Methodological considerations and study limitations

A central methodological contribution of this study is the explicit separation of cutting mortality from rooting failure. Wilson and Struve (2004) noted that computing rooting success from the initial cutting count confounds mortality with the failure of surviving tissue to root, and proposed separating survival proportion from rooting percentage among survivors. By modelling mortality as the primary event and right-censoring cuttings that survived to the final observation date, the Cox framework separates the temporal analysis of mortality from the assessment of rooting competence and recovers mortality patterns that endpoint summaries cannot resolve (Duarte et al., 2023; Lee, 2023). The value is clear in Stage 2. The endpoint analysis detected no significant DBH-group effect, whereas the time-to-event model identified a significant maternal DBH × IBA interaction. This indicates that the association between maternal DBH and mortality differed between IBA and the untreated control rather than operating as a uniform DBH effect across treatments. For protocol development, this pattern supports further testing of donor-size-specific auxin concentrations and exposure durations, rather than immediate adoption of a single reduced-auxin treatment for all larger-DBH donors.

Several methodological constraints should be acknowledged. First, the multivariable Cox model for Stage 2 retained only five of the twelve sampled genotypes owing to minimum sample size requirements per genotype-by-treatment cell. The remaining genotypes were analysed descriptively via Kaplan-Meier estimation, and the absence of significance for several genotype × auxin combinations should not be interpreted as evidence of absence but rather as reflecting the limited statistical resolution of the present design. Second, all Stage 2 propagation was conducted on peat-perlite, which precludes formal testing of substrate × genotype interactions despite their biological plausibility. Third, a marginal violation of the proportional hazards assumption was detected for the Genotype DG-15–6 main effect, suggesting that its baseline hazard may not remain constant over the full observation period (Lee, 2023). This covariate was retained because all associated interaction terms satisfied the proportionality assumption, but interpretation of its main effect coefficient should be treated with caution. Fourth, sample sizes for certain genotype-by-treatment cells were constrained by the natural availability of shoot material on wild donor trees. Notably, DG-15–9 under CK comprised only 3 cuttings. These small per-cell sizes reduce statistical power and may partially explain the non-significance of several interaction terms with biologically suggestive hazard ratio magnitudes.

Cutting size is a further source of heterogeneity that the present models do not address. Shoots were harvested at approximately 10 to 25 cm in both stages, and across that range the shortest and the longest cuttings within a treatment cell will have differed in leaf area, stem diameter and stored reserves. Individual cutting length was not measured or recorded, so it could not enter either Cox model as a covariate, and no analysis reported here separates its contribution from those of substrate, PGR treatment, maternal DBH or genotype. The individual-level time-to-event structure gives every cutting its own event time, but it does not adjust for cutting-level traits that were never recorded. The hazard ratios should therefore be read as averages over whatever length distribution each treatment cell happened to contain, not as estimates adjusted for cutting size. Cuttings were allocated to treatments without reference to length, so there is no reason to expect that distribution to differ systematically between cells, but that is an argument from the allocation procedure rather than something the present data can confirm. Recording length, basal diameter and leaf number for every cutting would let a future trial settle it.

Sex data were available for only 6 of the 33 sampled donors, precluding meaningful evaluation of sex-specific effects on cutting survival. This limitation is particularly relevant because sex-biased rooting capacities have been documented in other dioecious Taxaceae species, such as *T. baccata* (Alegre et al., 2001). The experimental material was drawn exclusively from the DAWU population. While DAWU harbours the most comprehensively documented individual-level census (Ho et al., 2012; Ko et al., 2025), transferability of propagation parameters to the Chachayalaishan (CHA) and Dalili (DL) populations cannot be assumed given the significant inter-population genetic differentiation documented by microsatellite analysis (Ko et al., 2025). The 15 DAWU management units represent a subset of the 29 genetic conservation units identified across the species’ entire range. The propagation responses of the CHA and DL genetic clusters remain uncharacterized. A further caveat applies to the smoothed hazard curves. Each panel of **Figure 3** is estimated within a single substrate-by-PGR cell, with a median of 8 cuttings percell, so the pointwise 95% confidence bands are wide relative to the point estimates (**Supplementary Figure 5**). These curves are therefore descriptive. They show when deaths clustered in time, and differences between individual curves should not be read as tests. Formal inference on substrate and PGR effects rests on the Cox model in **Table 3**, which pools all 252 cuttings and reports hazard ratios with 95% confidence intervals, andon the log-rank tests reported in the Results. Mortality was detected only at scheduled inspections,so the exact time of death within each inspection interval was unknown. Assigning the event to the date on which death was first confirmed may therefore slightly overestimate the actual survival time, although the common inspection schedule ensured that this temporal approximation was applied consistently across all treatment groups. Because that shift applies to every cutting through the same grid, it moves the time axis without altering the order in which deaths occurred, so it affects the dates attached to the Kaplan-Meier estimates rather than the hazard ratios. Refitting both Cox models with each event time replaced by the midpoint of its interval changes no coefficient by more than 3.886 × 10^-^¹^6^ in Stage 1 and 3.331 × 10^-^¹^6^ in Stage 2, which is machine rounding (**Supplementary Table 8**).

Two further limitations concern the estimation rather than the design. The ridge penalty thatStage 1 needs for identification also shapes what survives significance testing. Across penaliservalues from 0.01 to 0.5 the Substrate D hazard ratio stays significant, but the IBA main effect and the Substrate C × IBA interaction both lose nominal significance once the penalty reaches 0.2 (**Supplementary Table 11**), so those two auxin results are conditional on the chosen penalty. Stage 2 runs the other way, because both reported interactions are stronger without any penalty, which makes the values in **Table 4** the conservative ones. Second, under the fully interval-censored Weibull model the SubstrateC × IBA effect keeps its direction but loses significance (**Supplementary Table 9**). That is the one place where the two likelihoods disagree, and since the interaction restson a single cell of eight cuttings it should be treated as provisional. A 500-resample bootstrap ofthe Stage 2 model returns the same two findings, with the maternal DBH × IBA interaction above 1.000 in 99.00% of resamples and the DG-15-12 × NAA interaction below 1.000 in every resample (**Supplementary Table 10**).

Finally, the mechanisms proposed for the substrate- and PGR-dependent patterns are inferred from survival trajectories, not from direct measurement. Endogenous auxins were not quantified. Phenolic compounds, antioxidant status, and stress biomarkers were not measured. Monitoring ended once cuttings had survived the initial establishment phase. The hormonal and oxidative processes invoked above remain hypotheses and longer-term persistence is uncharacterised. The study is deliberately analytical in scope, quantifying how substrate, auxin, maternal ontogeny, and genotype interact to shape the timing and magnitude of cutting mortality. Four physiological questions that a survival analysis cannot resolve on its own define the next steps. First, histological staging of the induction, initiation, and expression phases, with expression profiling of rooting regulators such as the GRAS family and WOX11 (Wan et al., 2023), would link the mortality-timing signal to the underlying developmental programme. Second, time-resolved measurement of substrate physical and chemical properties, including compaction and dissolved oxygen, would test hydrological explanations suggested by the delayed rockwool mortality peak. Third, direct measurement of tissue auxin dynamics would test whether the observed timing of IBA-associated mortality is linked to early auxin induction, metabolism, or later stress responses, given that a brief quick-dip is buffered by conjugation and catabolism. Fourth, mineral nutrition, held at nursery-standard conditions here, is a further axis worth incorporating. These extensions, and longer post-establishment monitoring in the remaining populations, were beyond the present design. The experiment concluded in 2015 and the surviving cuttings were transferred to the Taitung Branch, Forestry and Nature Conservation Agency for *ex situ* conservation, which both precludes destructive re-sampling of this endangered species and represents a direct translation of the work into conservation practice. Despite these limitations, the study establishes the first quantitative, individual-level survival framework for propagation of any *Amentotaxus* species, revealing temporal patterns and factor-specific associations that were obscured by endpoint summaries of the severe post-rooting mortality reported for *A. formosana* (Chung et al., 2019), and it provides an empirical basis for genotype-matched, risk-informed propagation protocols.

## 5 Conclusions

This study is the first to model cutting mortality in *Amentotaxus formosana* as time-to-event data rather than as a single endpoint proportion. Kaplan-Meier estimation and Cox proportional hazards modelling separated when cuttings died from how many ultimately survived, and that separation showed that substrate type, PGR treatment, maternal DBH, and genotype contributed to mortality patterns through a combination of main effects and specific interactions. The framework recovers temporal and interaction structure that endpoint survival rates discard, and the same approach can be applied to other slow-rooting, recalcitrant woody species.

Three results from this trial are relevant to propagation practice. Pure shredded shale roughly doubled the mortality hazard relative to the peat-perlite mix, so free-draining shale-only media performed poorly during establishment under the conditions tested. IBA lowered mortality risk relative to ABT, but its interaction with maternal DBH indicates that a uniform high-concentration IBA protocol may not perform consistently across donor sizes. Lower concentrations, shorter exposure durations, or alternative auxins are worth testing for cuttings from larger-DBH donors. The genotype by auxin interaction detected for DG-15–12 under NAA further indicates that a single auxin treatment need not suit every donor genotype, so propagation trials for this species should be blocked by genotype rather than pooled. These three results should be read as evidence-based preliminary guidance rather than a validated propagation protocol. All come from a single nursery under one misting and shading regime, with no direct physiological measurement and individual cutting length uncontrolled. They should therefore be reproduced under independent nursery conditions and paired with direct physiological measurement before they are adopted as propagation recommendations. The trial has also had a direct conservation outcome: the surviving cuttings have already been transferred to the Taitung Branch, Forestry and Nature Conservation Agency for continued *ex situ* conservation of this endangered relict conifer.

## Data Availability

The original contributions presented in the study are included in the article/**Supplementary Material**. Further inquiries can be directed to the corresponding authors.
