# Supplementary material for: Maternal ontogeny modulates auxin-associated cutting mortality in Amentotaxus formosana, with genotype-specific responses revealed by Cox proportional hazards modelling
Source: Front Plant Sci. 2026 Aug 19;17:1892264. doi: 10.3389/fpls.2026.1892264 (PMC13533908; doi:10.3389/fpls.2026.1892264)
Supplement: Supplementary file 1 [file DataSheet2.docx]

Supplementary Material

# 1 Supplementary Methods

**S1.1 Site selection.** Although *Amentotaxus formosana* persists across three principal localities in Taiwan, sampling was restricted to the Dawu Taiwan Amentotaxus Nature Reserve (DAWU) for two reasons. First, DAWU is the only population for which a comprehensive, individual-level ecological census has been completed. Each recorded individual is documented with geo-referenced coordinates, diameter at breast height (DBH), tree height, basal stem diameter (BD), sex, and a standardized five-tier forest health index (Forestry Management Office, 2009; Ho et al., 2012), providing the individual-level resolution that the time-to-event framework adopted here requires. Second, the Chachayalaishan Major Wildlife Habitat, the other large-population locality of the species, is situated in geologically unstable terrain subject to frequent landslides, making systematic and repeated field access for cutting harvest operationally impracticable.

**S1.2 Donor screening and ontogenetic classification.** The five-tier forest health index applied to candidate donors (Ho et al., 2012) is anchored at 1 (moribund or dead) and 5 (vigorously growing), with grade 3 marking the onset of physiological deterioration. Grade 3 was set as the eligibility threshold so that baseline mortality attributable to poor donor condition would not be confounded with the treatment effects under test, and grades 4 and 5 were sampled preferentially wherever the shoot supply within a management unit allowed. This threshold is what excluded DG-15-5, DG-15-8, and DG-15-10 from the experiment, because every individual assigned to those three units scored 1 or 2 at the time of harvest. Ontogenetic interpretation of donor DBH rests on the demography of the species. *A. formosana* typically reaches 60 to 80 years of age, and individuals of 5 to 10 cm DBH are classified as middle-aged (Chan, 2005; Ko et al., 2025), so the 7.5 cm median used to stratify the 33 donors (Supplementary Table S1) falls at the midpoint of that band rather than at an arbitrary statistical boundary.

**S1.3 Analytical framework.** Cutting mortality in *A. formosana* unfolds across a multi-month pre-rooting period in which the timing, rate, and temporal clustering of deaths carry physiological information that is erased when the data are reduced to a single terminal proportion. Endpoint ANOVA was retained because it is the form in which almost all prior Taxaceae propagation results are reported, and dropping it would sever comparability with that literature. Kaplan-Meier estimation and Cox proportional hazards regression were added because they recover the part of the mortality process that a terminal proportion discards, in particular the pre-rooting window in which most deaths in this species occur. The same two-level pairing has been applied to slow-rooting forest species by Duarte et al. (2023) and to *ex situ* plant survival modelling by Thomas et al. (2022).

# 2 Supplementary Tables

**Supplementary Table S1.** Detailed biological characteristics of maternal source plants selected for cuttings in Stage 2 experiment.

| ID | Genotype | X-67 | Y-67 | DBH (cm) | Basal Diameter (cm) | Tree Height (m) | Sex |
| --- | --- | --- | --- | --- | --- | --- | --- |
| 133 | DG-15-1 | 225428.3 | 2477744 | 24.5 | 27.2 | 11.0 | F |
| 173 | DG-15-1 | 225419.1 | 2477766 | 11.5 | 15.8 | 9.2 | — |
| 114 | DG-15-2 | 225461.2 | 2477731 | 19.0 | 25.3 | 10.0 | F |
| 116 | DG-15-2 | 225462.3 | 2477731 | 14.5 | 18.0 | 5.5 | F |
| 119 | DG-15-2 | 225441.7 | 2477739 | 25.0 | 26.5 | 11.0 | M |
| 102 | DG-15-3 | 225465.3 | 2477705 | 2.0 | 2.5 | 1.3 | — |
| 104 | DG-15-3 | 225471.5 | 2477700 | 2.8 | 6.0 | 2.5 | — |
| 79 | DG-15-4 | 225421.0 | 2477694 | 2.0 | 6.0 | 1.7 | — |
| 108 | DG-15-4 | 225480.8 | 2477701 | 1.5 | 3.0 | 1.5 | — |
| 110 | DG-15-4 | 225476.7 | 2477714 | 1.2 | 2.0 | 1.8 | — |
| 211 | DG-15-6 | 225253.5 | 2477855 | 11.0 | 13.0 | 10.0 | — |
| 212 | DG-15-6 | 225253.4 | 2477835 | 13.0 | 20.0 | 12.0 | — |
| 213 | DG-15-6 | 225253.4 | 2477840 | 7.5 | 11.0 | 8.0 | — |
| 214 | DG-15-6 | 225249.3 | 2477833 | 11.0 | 15.0 | 11.0 | — |
| 54 | DG-15-7 | 225436.5 | 2477722 | 2.0 | 5.0 | 1.8 | — |
| 113 | DG-15-7 | 225463.3 | 2477716 | 1.5 | 2.0 | 2.0 | — |
| 151 | DG-15-7 | 225438.6 | 2477723 | 7.0 | 9.0 | 5.1 | — |
| 220 | DG-15-9 | 225469.5 | 2477732 | 30.0 | 35.0 | 15.0 | — |
| 225 | DG-15-9 | 225480.8 | 2477735 | 21.5 | 25.5 | 9.8 | F |
| 2 | DG-15-11 | 225470.6 | 2477816 | 4.0 | 6.0 | 2.1 | — |
| 25 | DG-15-11 | 225380.9 | 2477724 | 15.7 | 25.0 | 9.1 | M |
| 68 | DG-15-11 | 225396.3 | 2477713 | 6.5 | 7.0 | 3.8 | — |
| 69 | DG-15-12 | 225394.3 | 2477707 | 3.5 | 6.0 | 3.7 | — |
| 73 | DG-15-12 | 225382.9 | 2477686 | 7.5 | 11.0 | 5.3 | — |
| 111 | DG-15-12 | 225482.8 | 2477712 | 23.8 | 27.5 | 12.0 | — |
| 84 | DG-15-13 | 225440.6 | 2477700 | 4.3 | 6.3 | 4.2 | — |
| 85 | DG-15-13 | 225445.7 | 2477699 | 2.5 | 6.0 | 3.8 | — |
| 88 | DG-15-13 | 225455.0 | 2477705 | 20.0 | 22.0 | 15.0 | — |
| 11 | DG-15-14 | 225376.8 | 2477742 | 7.5 | 11.7 | 5.8 | — |
| 23 | DG-15-14 | 225376.8 | 2477744 | 4.5 | 7.0 | 6.2 | — |
| 44 | DG-15-15 | 225406.7 | 2477723 | 5.0 | 7.0 | 3.0 | — |
| 167 | DG-15-15 | 225407.8 | 2477763 | 14.0 | 16.5 | 7.0 | — |
| 169 | DG-15-15 | 225418.1 | 2477765 | 2.7 | 4.6 | 2.2 | — |

Notes: Genotype: DG-15-1 to DG-15-15 were identified and defined based on the assessment of genetic conservation units using microsatellite markers, as described in Ko et al. (2025). Sex: M, male; F, female; "—", unknown (due to juvenile stage or absence of reproductive strobili during the survey period). DBH: Diameter at Breast Height, measured at a standard height of 1.3 m above the ground level. Basal Diameter: Stem diameter measured at the root collar (ground level). X-67/Y-67: Spatial coordinates based on the TWD67 (Taiwan Datum 1967) projection system.

**Supplementary Table S2.** Two-way ANOVA (ordinary least squares, Type II sums of squares) of endpoint cutting survival in the Stage 1 and Stage 2 experiments.

| Experiment Stage | Source of Variation | Sum of Squares | *df* | *F*-value | *p*-value |
| --- | --- | --- | --- | --- | --- |
| Stage 1 (PGR & Substrate) | PGR (Agent) | 1.152 | 4 | 1.460 | 0.215 |
|  | Substrate | 3.095 | 4 | 3.923 | **0.004**** |
|  | PGR × Substrate | 6.747 | 16 | 2.138 | **0.008**** |
|  | Residual | 44.779 | 227 |  |  |
| Stage 2 (DBH & PGR) | Maternal DBH Group | 1196.277 | 1 | 3.479 | 0.069 |
|  | PGR (Treatment) | 785.851 | 2 | 1.143 | 0.328 |
|  | DBH × PGR | 247.415 | 2 | 0.360 | 0.700 |
|  | Residual | 15475.035 | 45 |  |  |

Note: Both stages were analysed by ordinary least squares with Type II sums of squares, but not on the same scale. In Stage 1, final cutting survival (1 = alive, 0 = dead) at the final observation day was modelled at the individual cutting level (n = 252), with PGR treatment, substrate, and their interaction as fixed effects. This is a linear probability model, which provides unbiased estimates of average marginal effects for binary outcomes and avoids the complete separation problem encountered when cell survival proportions reach 0% or 100%, which precludes convergence of maximum likelihood estimation in logistic regression. In Stage 2, cell sizes were unequal across genotype-by-treatment combinations, so final survival was aggregated to genotype × DBH group × PGR cells (n = 51 effective cells) and the model was fitted to those cell percentages, with DBH group, PGR treatment, and their interaction as fixed effects. Each percentage is the pooled rate over the donor trees contributing to the cell. The sums of squares of the two stages are therefore not comparable with each other. Significance threshold: α = 0.05. Primary inferences on treatment effects are drawn from the Cox proportional hazards model (Table 3 and Table 4).

**Supplementary Table S3.** Endpoint cutting survival rate (mean percentage ± SE) for each substrate-by-PGR combination in the Stage 1 experiment.

| PGR Treatment | Substrate A | Substrate B | Substrate C | Substrate D | Substrate E |
| --- | --- | --- | --- | --- | --- |
| CK (Control) | 71.43 ± 18.44 ^abc^ | 33.33 ± 16.67 ^abc^ | 50.00 ± 18.90 ^abc^ | 29.41 ± 11.39 ^abc^ | 0.00 ± 0.00 ^bc^ |
| NAA | 62.50 ± 18.30 ^abc^ | 50.00 ± 18.90 ^abc^ | 28.57 ± 18.44 ^abc^ | 16.67 ± 11.24 ^abc^ | 9.09 ± 9.09 ^bc^ |
| IBA | 87.50 ± 12.50 ^a^ | 44.44 ± 17.57 ^abc^ | 25.00 ± 16.37 ^abc^ | 30.00 ± 10.51 ^abc^ | 36.36 ± 15.21 ^abc^ |
| IAA | 37.50 ± 18.30 ^abc^ | 37.50 ± 18.30 ^abc^ | 25.00 ± 16.37 ^abc^ | 35.71 ± 13.29 ^abc^ | 35.71 ± 13.29 ^abc^ |
| ABT | 12.50 ± 12.50 ^abc^ | 0.00 ± 0.00 ^bc^ | 75.00 ± 16.37 ^ab^ | 0.00 ± 0.00 ^c^ | 36.36 ± 15.21 ^abc^ |

Substrate codes are A (peat-perlite), B (shale-akadama mix), C (coconut-fibre mix), D (pure shredded shale), and E (rockwool).

Note: Data are presented as mean percentages ± standard error (SE). Different superscript letters indicate significant differences between treatment combinations according to Tukey’s HSD post-hoc test (*p* < 0.05). Endpoint survival is scored at the final inspection on day 516, the last of the 14 scheduled Stage 1 inspection dates.

**Supplementary Table S4.** Cumulative mortality of *A. formosana* cuttings at selected observation time points across five rooting substrate conditions in the Stage 1 experiment (26 September 2013 to 24 February 2015). For each substrate, total cuttings, total death events, and cumulative death counts (n) and percentages (%) are presented at eight reference time points spanning the 516-day observation period. Percentages are calculated as cumulative deaths relative to the total number of cuttings in each substrate group, including survivors, successfully rooted cuttings, and right-censored observations. Values at Day 516 below 100% therefore reflect cuttings that did not die during the observation period. The row "All substrates combined" pools all 252 cuttings across the five substrate conditions.

| Substrate | Total cuttings (n) | Total deaths (n) | Cumulative deaths, *n* (% of total cuttings) | | | | | | | |
| --- | --- | --- | --- | --- | --- | --- | --- | --- | --- | --- |
|  |  |  | Day 30 | Day 54 | Day 92 | Day 126 | Day 191 | Day 237 | Day 360 | Day 516 |
| Substrate A | 39 | 18 | 5 (12.82) | 8 (20.51) | 10 (25.64) | 11 (28.21) | 11 (28.21) | 11 (28.21) | 15 (38.46) | 18 (46.15) |
| Substrate B | 42 | 28 | 6 (14.29) | 9 (21.43) | 19 (45.24) | 25 (59.52) | 25 (59.52) | 25 (59.52) | 26 (61.90) | 28 (66.67) |
| Substrate C | 39 | 23 | 5 (12.82) | 8 (20.51) | 12 (30.77) | 17 (43.59) | 17 (43.59) | 17 (43.59) | 21 (53.85) | 23 (58.97) |
| Substrate D | 78 | 60 | 22 (28.21) | 32 (41.03) | 50 (64.10) | 55 (70.51) | 55 (70.51) | 56 (71.79) | 60 (76.92) | 60 (76.92) |
| Substrate E | 54 | 40 | 0 (0.00) | 14 (25.93) | 27 (50.00) | 29 (53.70) | 39 (72.22) | 39 (72.22) | 39 (72.22) | 40 (74.07) |
| All substrates combined | 252 | 169 | 38 (15.08) | 71 (28.17) | 118 (46.83) | 137 (54.37) | 147 (58.33) | 148 (58.73) | 161 (63.89) | 169 (67.06) |

Substrate codes are A (peat-perlite), B (shale-akadama mix), C (coconut-fibre mix), D (pure shredded shale), and E (rockwool).

Notes: Cumulative deaths are counted from event times defined as the scheduled inspection date at which death was first confirmed. Seven of the eight columns are scheduled inspection dates. Day 360 is a reference readout point and not an inspection date, so the counts in that column are those accumulated through the last inspection on or before it, on day 353. The full Stage 1 inspection schedule is 30, 54, 92, 126, 191, 237, 298, 330, 353, 383, 413, 448, 494 and 516 days.

**Supplementary Table S5.** Kaplan-Meier survival estimates for suboptimal substrate and PGR combinations (Stage 1 experiment).

| Suboptimal Combination | Day 92 (%) | Day 191 (%) | Day 360 (%) |
| --- | --- | --- | --- |
| Substrate C + IAA | 62.50 | 37.50 | 37.50 |
| Substrate C + IBA | 50.00 | 50.00 | 37.50 |
| Substrate B + IAA | 37.50 | 37.50 | 37.50 |
| Substrate E + IBA | 45.45 | 36.36 | 36.36 |
| Substrate E + ABT | 45.45 | 36.36 | 36.36 |
| Substrate D + IAA | 50.00 | 50.00 | 35.71 |
| Substrate E + IAA | 64.29 | 35.71 | 35.71 |
| Substrate B + CK | 77.78 | 33.33 | 33.33 |
| Substrate D + IBA | 40.00 | 40.00 | 30.00 |
| Substrate D + CK | 47.06 | 29.41 | 29.41 |
| Substrate C + NAA | 57.14 | 42.86 | 28.57 |
| Substrate A + ABT | 37.50 | 37.50 | 25.00 |
| Substrate E + NAA | 36.36 | 18.18 | 18.18 |
| Substrate D + NAA | 25.00 | 16.67 | 16.67 |
| Substrate B + ABT | 12.50 | 12.50 | 0.00 |
| Substrate D + ABT | 13.33 | 6.67 | 0.00 |
| Substrate E + CK | 57.14 | 0.00 | 0.00 |

Substrate codes are A (peat-perlite), B (shale-akadama mix), C (coconut-fibre mix), D (pure shredded shale), and E (rockwool).

Notes: The table covers the 17 of the 25 substrate-by-PGR cells whose day-360 Kaplan-Meier survival fell below 50.00%, listed in descending order of that value. Cell sizes range from 7 to 20 cuttings, so every estimate rests on a single small cell. Survival is computed from event times defined as the scheduled inspection date at which death was first confirmed. Day 360 is a reference readout point and not an inspection date, so the values in that column are those carried forward from the last inspection on or before it, on day 353.

**Supplementary Table S6.** Pairwise log-rank test results for all 66 genotype combinations in the Stage 2 experiment. Pairwise comparisons were conducted on data pooled across all three treatment conditions (CK, IBA, and NAA). Genotype pairs are sorted by *p*-value in ascending order. Rows highlighted in yellow indicate statistically significant differences (*p* < 0.05). The "Higher survival" column indicates the genotype with higher Kaplan-Meier estimated survival probability at day 374 (S(374)) in each significant pair, determined from survival data pooled across all treatment conditions. "—" indicates no statistically significant difference. Significance codes: *** *p* < 0.001; ** *p* < 0.01; * *p* < 0.05; ns, not significant (*p* ≥ 0.05).

| Genotype A | Genotype B | *χ²* | *p*-value | Sig. | Higher survival |
| --- | --- | --- | --- | --- | --- |
| DG-15-4 | DG-15-6 | 32.112 | < 0.001 | ******* | DG-15-4 |
| DG-15-4 | DG-15-9 | 31.526 | < 0.001 | ******* | DG-15-4 |
| DG-15-1 | DG-15-9 | 25.535 | < 0.001 | ******* | DG-15-1 |
| DG-15-1 | DG-15-6 | 23.513 | < 0.001 | ******* | DG-15-1 |
| DG-15-4 | DG-15-7 | 18.608 | < 0.001 | ******* | DG-15-4 |
| DG-15-3 | DG-15-9 | 17.686 | < 0.001 | ******* | DG-15-3 |
| DG-15-3 | DG-15-6 | 15.933 | < 0.001 | ******* | DG-15-3 |
| DG-15-9 | DG-15-14 | 13.055 | < 0.001 | ******* | DG-15-14 |
| DG-15-1 | DG-15-7 | 11.512 | < 0.001 | ******* | DG-15-1 |
| DG-15-9 | DG-15-11 | 11.055 | < 0.001 | ******* | DG-15-11 |
| DG-15-4 | DG-15-11 | 11.043 | < 0.001 | ******* | DG-15-4 |
| DG-15-9 | DG-15-15 | 10.231 | 0.001 | ****** | DG-15-15 |
| DG-15-6 | DG-15-14 | 9.721 | 0.002 | ****** | DG-15-14 |
| DG-15-2 | DG-15-4 | 9.581 | 0.002 | ****** | DG-15-4 |
| DG-15-4 | DG-15-13 | 9.407 | 0.002 | ****** | DG-15-4 |
| DG-15-3 | DG-15-7 | 8.701 | 0.003 | ****** | DG-15-3 |
| DG-15-6 | DG-15-15 | 8.446 | 0.004 | ****** | DG-15-15 |
| DG-15-9 | DG-15-12 | 8.201 | 0.004 | ****** | DG-15-12 |
| DG-15-6 | DG-15-11 | 7.864 | 0.005 | ****** | DG-15-11 |
| DG-15-4 | DG-15-12 | 7.420 | 0.006 | ****** | DG-15-4 |
| DG-15-1 | DG-15-11 | 7.209 | 0.007 | ****** | DG-15-1 |
| DG-15-4 | DG-15-15 | 7.177 | 0.007 | ****** | DG-15-4 |
| DG-15-9 | DG-15-13 | 6.905 | 0.009 | ****** | DG-15-13 |
| DG-15-6 | DG-15-12 | 6.515 | 0.011 | ***** | DG-15-12 |
| DG-15-1 | DG-15-2 | 5.478 | 0.019 | ***** | DG-15-1 |
| DG-15-1 | DG-15-13 | 5.316 | 0.021 | ***** | DG-15-1 |
| DG-15-7 | DG-15-14 | 4.734 | 0.030 | ***** | DG-15-14 |
| DG-15-7 | DG-15-11 | 4.657 | 0.031 | ***** | DG-15-11 |
| DG-15-6 | DG-15-13 | 4.572 | 0.032 | ***** | DG-15-13 |
| DG-15-7 | DG-15-15 | 4.280 | 0.039 | ***** | DG-15-15 |
| DG-15-2 | DG-15-9 | 4.239 | 0.039 | ***** | DG-15-2 |
| DG-15-1 | DG-15-12 | 4.012 | 0.045 | ***** | DG-15-1 |
| DG-15-1 | DG-15-15 | 3.897 | 0.048 | ***** | DG-15-1 |
| DG-15-2 | DG-15-3 | 3.727 | 0.054 | ns | — |
| DG-15-3 | DG-15-11 | 3.551 | 0.060 | ns | — |
| DG-15-3 | DG-15-13 | 3.379 | 0.066 | ns | — |
| DG-15-4 | DG-15-14 | 3.196 | 0.074 | ns | — |
| DG-15-7 | DG-15-12 | 3.069 | 0.080 | ns | — |
| DG-15-2 | DG-15-6 | 2.584 | 0.108 | ns | — |
| DG-15-7 | DG-15-13 | 2.355 | 0.125 | ns | — |
| DG-15-3 | DG-15-12 | 2.115 | 0.146 | ns | — |
| DG-15-3 | DG-15-15 | 1.820 | 0.177 | ns | — |
| DG-15-2 | DG-15-14 | 1.452 | 0.228 | ns | — |
| DG-15-7 | DG-15-9 | 1.436 | 0.231 | ns | — |
| DG-15-3 | DG-15-4 | 1.379 | 0.240 | ns | — |
| DG-15-2 | DG-15-7 | 1.328 | 0.249 | ns | — |
| DG-15-1 | DG-15-14 | 1.244 | 0.265 | ns | — |
| DG-15-6 | DG-15-9 | 1.069 | 0.301 | ns | — |
| DG-15-13 | DG-15-14 | 0.978 | 0.323 | ns | — |
| DG-15-2 | DG-15-15 | 0.864 | 0.353 | ns | — |
| DG-15-11 | DG-15-14 | 0.831 | 0.362 | ns | — |
| DG-15-2 | DG-15-11 | 0.661 | 0.416 | ns | — |
| DG-15-12 | DG-15-14 | 0.589 | 0.443 | ns | — |
| DG-15-1 | DG-15-3 | 0.577 | 0.448 | ns | — |
| DG-15-3 | DG-15-14 | 0.424 | 0.515 | ns | — |
| DG-15-13 | DG-15-15 | 0.421 | 0.517 | ns | — |
| DG-15-2 | DG-15-12 | 0.339 | 0.561 | ns | — |
| DG-15-14 | DG-15-15 | 0.191 | 0.662 | ns | — |
| DG-15-1 | DG-15-4 | 0.185 | 0.667 | ns | — |
| DG-15-6 | DG-15-7 | 0.175 | 0.676 | ns | — |
| DG-15-11 | DG-15-13 | 0.125 | 0.723 | ns | — |
| DG-15-11 | DG-15-15 | 0.118 | 0.731 | ns | — |
| DG-15-2 | DG-15-13 | 0.112 | 0.738 | ns | — |
| DG-15-12 | DG-15-15 | 0.094 | 0.759 | ns | — |
| DG-15-12 | DG-15-13 | 0.091 | 0.763 | ns | — |
| DG-15-11 | DG-15-12 | 0.000 | 0.983 | ns | — |

**Notes:** S(374) denotes the Kaplan-Meier estimated survival probability at the end of the 374-day observation period, pooled across all three treatment conditions (CK, IBA, and NAA) within each genotype. Values for all 12 genotypes: DG-15-4 (0.400), DG-15-3 (0.292), DG-15-1 (0.286), DG-15-14 (0.227), DG-15-15 (0.211), DG-15-12 (0.189), DG-15-2 (0.182), DG-15-13 (0.167), DG-15-11 (0.132), DG-15-7 (0.088), DG-15-6 (0.038), DG-15-9 (0.000). DG-15-4 showed significantly higher survival than 8 other genotypes (DG-15-2, DG-15-6, DG-15-7, DG-15-9, DG-15-11, DG-15-12, DG-15-13, and DG-15-15, all *p* < 0.05). DG-15-9 and DG-15-6 showed significantly lower survival than 9 and 8 other genotypes, respectively (all *p* < 0.05).

**Supplementary Table S7.** Assessment of the Proportional Hazards (PH) assumption using scaled Schoenfeld residuals. The table has two panels, the Stage 1 substrate × PGR model with 24 terms and the Stage 2 maternal DBH and genotype model with 17.

| Covariate | Test Statistic (*χ*2) | *df* | *p*-value |
| --- | --- | --- | --- |
| Stage 1 model: substrate × PGR, 24 terms |  |  |  |
| Main Effects |  |  |  |
| CK vs ABT | 1.329 | 1 | 0.249 |
| IAA vs ABT | 1.839 | 1 | 0.175 |
| IBA vs ABT | 0.579 | 1 | 0.447 |
| NAA vs ABT | 2.417 | 1 | 0.120 |
| Substrate B | 0.226 | 1 | 0.635 |
| Substrate C | 0.124 | 1 | 0.724 |
| Substrate D | 0.461 | 1 | 0.497 |
| Substrate E | 0.808 | 1 | 0.369 |
| Interaction Effects |  |  |  |
| Substrate B × CK | 1.468 | 1 | 0.226 |
| Substrate B × IAA | 0.016 | 1 | 0.901 |
| Substrate B × IBA | 0.987 | 1 | 0.320 |
| Substrate B × NAA | 0.391 | 1 | 0.532 |
| Substrate C × CK | 0.334 | 1 | 0.563 |
| Substrate C × IAA | 1.536 | 1 | 0.215 |
| Substrate C × IBA | 0.444 | 1 | 0.505 |
| Substrate C × NAA | 0.017 | 1 | 0.896 |
| Substrate D × CK | 0.932 | 1 | 0.334 |
| Substrate D × IAA | 0.138 | 1 | 0.710 |
| Substrate D × IBA | 0.008 | 1 | 0.927 |
| Substrate D × NAA | 0.081 | 1 | 0.776 |
| Substrate E × CK | 2.183 | 1 | 0.140 |
| Substrate E × IAA | 0.193 | 1 | 0.660 |
| Substrate E × IBA | 0.062 | 1 | 0.803 |
| Substrate E × NAA | 0.040 | 1 | 0.841 |
| Stage 2 model: maternal DBH and genotype × auxin, 17 terms |  |  |  |
| Main Effects |  |  |  |
| DBH | 0.008 | 1 | 0.930 |
| IBA | 0.441 | 1 | 0.507 |
| NAA | 0.612 | 1 | 0.434 |
| Genotype DG-15-6 | 5.354 | 1 | 0.021* |
| Genotype DG-15-12 | 0.528 | 1 | 0.467 |
| Genotype DG-15-13 | 0.710 | 1 | 0.399 |
| Genotype DG-15-15 | 0.064 | 1 | 0.800 |
| Interaction Effects |  |  |  |
| Maternal DBH × IBA | 0.173 | 1 | 0.678 |
| Maternal DBH × NAA | 0.784 | 1 | 0.376 |
| Genotype DG-15-6 × IBA | 0.044 | 1 | 0.833 |
| Genotype DG-15-6 × NAA | 0.095 | 1 | 0.757 |
| Genotype DG-15-12 × IBA | 1.407 | 1 | 0.236 |
| Genotype DG-15-12 × NAA | 0.451 | 1 | 0.502 |
| Genotype DG-15-13 × IBA | 2.018 | 1 | 0.155 |
| Genotype DG-15-13 × NAA | 0.342 | 1 | 0.558 |
| Genotype DG-15-15 × IBA | 0.008 | 1 | 0.927 |
| Genotype DG-15-15 × NAA | 0.182 | 1 | 0.670 |

Notes: The null hypothesis of the Schoenfeld residuals test is that the hazard ratio is constant over time, and *p* > 0.05 indicates that the proportional hazards assumption is met. None of the 24 Stage 1 terms departed from proportionality at α = 0.05, against 1.2 expected by chance at that threshold, and 1 of the 17 Stage 2 terms did, against 0.9 expected. The rank-transform test served as the primary assessment. Under this test, only the Genotype DG-15-6 main effect showed a marginal violation (*p* = 0.021). A km-transform test additionally flagged Genotype DG-15-13 × IBA (*p* = 0.037), but the rank-based test for the same term was non-significant (*p* = 0.155), so it is not treated as a violation. Genotype DG-15-6 was retained as a necessary structural term because all of its associated interaction terms satisfied the proportionality assumption, and this marginal violation is acknowledged as a limitation on the direct interpretation of its main-effect coefficient.

**Supplementary Table S8.** Cox estimates under two event-time conventions, the right endpoint of the inspection interval and the interval midpoint. The two event-time conventions produce the same estimates to within rounding error. The largest absolute difference in any coefficient is 3.886 × 10⁻¹⁶ in Stage 1 and 3.331 × 10⁻¹⁶ in Stage 2.

| Model term | Right endpoint HR | Right endpoint p | Midpoint HR | Midpoint p |
| --- | --- | --- | --- | --- |
| Stage 1 model |  |  |  |  |
| Substrate D | 2.338 | 0.001 | 2.338 | 0.001 |
| IBA vs ABT | 0.517 | 0.027 | 0.517 | 0.027 |
| Substrate C × IBA | 3.066 | 0.037 | 3.066 | 0.037 |
| Stage 2 model |  |  |  |  |
| Maternal DBH per SD | 1.050 | 0.699 | 1.050 | 0.699 |
| IBA | 0.774 | 0.299 | 0.774 | 0.299 |
| NAA | 0.989 | 0.964 | 0.989 | 0.964 |
| Maternal DBH × IBA | 1.388 | 0.048 | 1.388 | 0.048 |
| Maternal DBH × NAA | 1.298 | 0.120 | 1.298 | 0.120 |
| Genotype DG-15-12 × NAA | 0.386 | 0.035 | 0.386 | 0.035 |

Notes: Deaths were detected at a fixed schedule of inspections shared by every cutting, so the recorded event time is the right endpoint of the interval in which death occurred. The Cox partial likelihood is a function of the rank order of the event times alone. Replacing each recorded time by the midpoint of its interval is a strictly increasing relabelling of a grid common to the whole experiment, so every risk set is unchanged and every coefficient is unchanged with it. The largest absolute difference in any fitted coefficient was 3.886 × 10⁻¹⁶ in Stage 1 and 3.331 × 10⁻¹⁶ in Stage 2, which is machine rounding rather than a numerical difference. Both fits use a ridge penalizer of 0.1 and Efron’s approximation for ties. HR, hazard ratio.

**Supplementary Table S9.** Weibull accelerated failure time models fitted under the exact interval-censored likelihood, set against the Cox estimates reported in Tables 3 and 4. No event time is imputed. An accelerated failure time coefficient below zero shortens survival and so corresponds to a hazard ratio above one.

| Model term | AFT coefficient | AFT p | Cox HR | Cox p | Comparison |
| --- | --- | --- | --- | --- | --- |
| Stage 1 model |  |  |  |  |  |
| Substrate D | -0.940 | 0.002 | 2.338 | 0.001 | Same direction, same call |
| IBA vs ABT | 0.665 | 0.046 | 0.517 | 0.027 | Same direction, same call |
| Substrate C × IBA | -1.015 | 0.129 | 3.066 | 0.037 | Same direction, different call |
| Stage 2 model |  |  |  |  |  |
| Maternal DBH per SD | -0.103 | 0.461 | 1.050 | 0.699 | Same direction, same call |
| IBA | 0.222 | 0.414 | 0.774 | 0.299 | Same direction, same call |
| NAA | -0.030 | 0.914 | 0.989 | 0.964 | Opposite direction, same call |
| Maternal DBH × IBA | -0.408 | 0.037 | 1.388 | 0.048 | Same direction, same call |
| Maternal DBH × NAA | -0.264 | 0.168 | 1.298 | 0.120 | Same direction, same call |
| Genotype DG-15-12 × NAA | 1.148 | 0.021 | 0.386 | 0.035 | Same direction, same call |

Notes: Each death contributes the probability of dying between the previous inspection and the inspection that confirmed it, and each survivor contributes a right-censored term. No event time is imputed, so this fit does not depend on the choice of event-time convention. AFT coefficients act on log survival time, so a negative coefficient means shorter survival and points the same way as a Cox hazard ratio above 1. Seven of the nine terms match the Cox result in both direction and significance call. The Stage 2 NAA main effect reverses sign, but neither estimate is distinguishable from no effect (*p* = 0.964 and *p* = 0.914). The Substrate C × IBA interaction keeps its direction and loses significance, at *p* = 0.129 against *p* = 0.037 under the Cox model. That interaction rests on a single cell of 8 cuttings and is treated as provisional in the text. AFT, accelerated failure time. HR, hazard ratio.

**Supplementary Table S10.** Stability of the Stage 2 Cox model. The upper panel gives the genotype-by-treatment cell counts and the lower panel the nonparametric bootstrap distribution of the reported terms over 500 resamples of cuttings with replacement.

| Genotype × treatment cell | Cuttings | Deaths | Mortality (%) |
| --- | --- | --- | --- |
| DG-15-11 (reference) × CK | 7 | 6 | 85.71 |
| DG-15-11 (reference) × IBA | 15 | 12 | 80.00 |
| DG-15-11 (reference) × NAA | 16 | 15 | 93.75 |
| DG-15-12 × CK | 6 | 6 | 100.00 |
| DG-15-12 × IBA | 16 | 16 | 100.00 |
| DG-15-12 × NAA | 15 | 8 | 53.33 |
| DG-15-13 × CK | 7 | 6 | 85.71 |
| DG-15-13 × IBA | 15 | 12 | 80.00 |
| DG-15-13 × NAA | 14 | 12 | 85.71 |
| DG-15-15 × CK | 8 | 7 | 87.50 |
| DG-15-15 × IBA | 15 | 11 | 73.33 |
| DG-15-15 × NAA | 15 | 12 | 80.00 |
| DG-15-6 × CK | 12 | 11 | 91.67 |
| DG-15-6 × IBA | 20 | 20 | 100.00 |
| DG-15-6 × NAA | 20 | 19 | 95.00 |
| Model term | Median HR | Bootstrap 95% CI | P(HR > 1) |
| Maternal DBH per SD | 1.070 | 0.914 – 1.276 | 0.786 |
| IBA | 0.756 | 0.537 – 1.028 | 0.040 |
| NAA | 0.980 | 0.704 – 1.384 | 0.460 |
| Maternal DBH × IBA | 1.386 | 1.103 – 1.693 | 0.990 |
| Maternal DBH × NAA | 1.292 | 0.958 – 1.737 | 0.956 |
| Genotype DG-15-12 × NAA | 0.370 | 0.178 – 0.709 | 0.000 |

Notes: Events per parameter were 7.04 in Stage 1, from 169 deaths among 252 cuttings across 24 parameters, and 10.18 in Stage 2, from 173 deaths among 201 cuttings across 17 parameters. Three of the 15 Stage 2 cells lost every cutting, which is what makes the ridge penalty necessary. The bootstrap drew 500 resamples of cuttings with replacement and refitted the same penalized model to each, and all 500 converged. Because every resample carries the same penalty of 0.1, these intervals describe how reproducible the reported estimates are under resampling. They are not unpenalized confidence intervals and are not offered as such. P(HR > 1) is the proportion of resamples in which the hazard ratio exceeded 1. The resampling is seeded, so the values are reproducible from the deposited script. HR, hazard ratio. CI, confidence interval.

**Supplementary Table S11.** Hazard ratios for the reported terms of both Cox models across ridge penalizer values from 0 to 1.

| Penalizer | Model term | HR | 95% CI | p |
| --- | --- | --- | --- | --- |
| Stage 1 model: substrate × PGR |  |  |  |  |
| 0 | Substrate D | 2.290 | 0.928 – 5.652 | 0.072 |
|  | IBA vs ABT | 0.062 | 0.008 – 0.508 | 0.010* |
|  | Substrate C × IBA | 80.262 | 5.741 – 1122.076 | 0.001* |
| 0.01 | Substrate D | 3.128 | 1.407 – 6.956 | 0.005* |
|  | IBA vs ABT | 0.196 | 0.058 – 0.663 | 0.009* |
|  | Substrate C × IBA | 16.574 | 2.926 – 93.896 | 0.002* |
| 0.05 | Substrate D | 2.856 | 1.568 – 5.203 | 0.001* |
|  | IBA vs ABT | 0.401 | 0.192 – 0.841 | 0.016* |
|  | Substrate C × IBA | 4.916 | 1.446 – 16.715 | 0.011* |
| 0.10 | Substrate D | 2.338 | 1.405 – 3.891 | 0.001* |
|  | IBA vs ABT | 0.517 | 0.289 – 0.926 | 0.027* |
|  | Substrate C × IBA | 3.066 | 1.069 – 8.793 | 0.037* |
| 0.20 | Substrate D | 1.846 | 1.219 – 2.796 | 0.004* |
|  | IBA vs ABT | 0.644 | 0.408 – 1.016 | 0.059 |
|  | Substrate C × IBA | 2.067 | 0.836 – 5.110 | 0.116 |
| 0.50 | Substrate D | 1.422 | 1.056 – 1.916 | 0.021* |
|  | IBA vs ABT | 0.795 | 0.575 – 1.101 | 0.167 |
|  | Substrate C × IBA | 1.435 | 0.701 – 2.941 | 0.323 |
| 1.00 | Substrate D | 1.245 | 0.992 – 1.562 | 0.058 |
|  | IBA vs ABT | 0.876 | 0.684 – 1.123 | 0.296 |
|  | Substrate C × IBA | 1.214 | 0.683 – 2.158 | 0.509 |
| Stage 2 model: maternal DBH and genotype × auxin |  |  |  |  |
| 0 | Maternal DBH per SD | 0.776 | 0.505 – 1.190 | 0.245 |
|  | IBA | 0.564 | 0.202 – 1.571 | 0.273 |
|  | NAA | 0.975 | 0.362 – 2.627 | 0.960 |
|  | Maternal DBH × IBA | 1.990 | 1.202 – 3.294 | 0.007* |
|  | Maternal DBH × NAA | 1.806 | 1.086 – 3.003 | 0.023* |
|  | Genotype DG-15-12 × NAA | 0.145 | 0.034 – 0.618 | 0.009* |
| 0.01 | Maternal DBH per SD | 0.852 | 0.579 – 1.252 | 0.414 |
|  | IBA | 0.614 | 0.270 – 1.398 | 0.245 |
|  | NAA | 1.004 | 0.449 – 2.242 | 0.993 |
|  | Maternal DBH × IBA | 1.792 | 1.131 – 2.840 | 0.013* |
|  | Maternal DBH × NAA | 1.635 | 1.026 – 2.605 | 0.039* |
|  | Genotype DG-15-12 × NAA | 0.181 | 0.049 – 0.666 | 0.010* |
| 0.05 | Maternal DBH per SD | 0.990 | 0.737 – 1.331 | 0.949 |
|  | IBA | 0.709 | 0.396 – 1.270 | 0.248 |
|  | NAA | 1.001 | 0.559 – 1.794 | 0.996 |
|  | Maternal DBH × IBA | 1.502 | 1.035 – 2.180 | 0.032* |
|  | Maternal DBH × NAA | 1.389 | 0.952 – 2.027 | 0.089 |
|  | Genotype DG-15-12 × NAA | 0.298 | 0.105 – 0.845 | 0.023* |
| 0.10 | Maternal DBH per SD | 1.050 | 0.820 – 1.343 | 0.699 |
|  | IBA | 0.774 | 0.476 – 1.256 | 0.299 |
|  | NAA | 0.989 | 0.606 – 1.615 | 0.964 |
|  | Maternal DBH × IBA | 1.388 | 1.003 – 1.922 | 0.048* |
|  | Maternal DBH × NAA | 1.298 | 0.934 – 1.802 | 0.120 |
|  | Genotype DG-15-12 × NAA | 0.386 | 0.159 – 0.936 | 0.035* |
| 0.20 | Maternal DBH per SD | 1.089 | 0.892 – 1.329 | 0.404 |
|  | IBA | 0.845 | 0.569 – 1.255 | 0.404 |
|  | NAA | 0.978 | 0.653 – 1.463 | 0.913 |
|  | Maternal DBH × IBA | 1.299 | 0.983 – 1.716 | 0.066 |
|  | Maternal DBH × NAA | 1.230 | 0.930 – 1.627 | 0.146 |
|  | Genotype DG-15-12 × NAA | 0.485 | 0.235 – 1.001 | 0.050 |
| 0.50 | Maternal DBH per SD | 1.102 | 0.952 – 1.276 | 0.192 |
|  | IBA | 0.925 | 0.689 – 1.241 | 0.601 |
|  | NAA | 0.972 | 0.722 – 1.310 | 0.854 |
|  | Maternal DBH × IBA | 1.206 | 0.968 – 1.502 | 0.095 |
|  | Maternal DBH × NAA | 1.166 | 0.939 – 1.448 | 0.165 |
|  | Genotype DG-15-12 × NAA | 0.615 | 0.360 – 1.050 | 0.075 |
| 1.00 | Maternal DBH per SD | 1.089 | 0.973 – 1.220 | 0.139 |
|  | IBA | 0.962 | 0.766 – 1.209 | 0.741 |
|  | NAA | 0.974 | 0.773 – 1.227 | 0.823 |
|  | Maternal DBH × IBA | 1.146 | 0.960 – 1.369 | 0.132 |
|  | Maternal DBH × NAA | 1.125 | 0.946 – 1.338 | 0.182 |
|  | Genotype DG-15-12 × NAA | 0.710 | 0.469 – 1.076 | 0.107 |

Notes: The value used throughout the paper is 0.1. In Stage 1 the penalty is needed for identification. Three substrate-by-PGR cells lost every cutting and a fourth lost 1 of 8 against a reference cell that lost 7 of 8, so the unpenalized partial likelihood has no finite maximiser for the affected contrasts. At a penalizer of 0 the Substrate C × IBA coefficient reaches 4.385, a hazard ratio of 80.262, with a standard error of 1.346 and a 95% confidence interval running from 5.741 to 1122.076. That is the signature of a coefficient drifting toward infinity rather than an estimate. The Substrate D effect is the stable one. Its hazard ratio moves only from 2.290 in the unpenalized fit to 2.338 at the reported value, so the penalty narrows its interval rather than shifting its estimate, and the term is nominally significant at every penalizer from 0.01 to 0.5. It falls just short at the two ends, at p = 0.072 in the degenerate unpenalized fit and p = 0.058 at a penalizer of 1, where the shrinkage has pulled every term toward the null. The IBA main effect and the Substrate C × IBA interaction behave differently, and both lose nominal significance once the penalty reaches 0.2, so both should be read as conditional on the chosen value. Stage 2 behaves in the opposite way. Its unpenalized fit is estimable, and both reported interactions are larger and more strongly supported without a penalty, so the values in Table 4 are the conservative ones. An asterisk marks *p* < 0.05. HR, hazard ratio. CI, confidence interval.

# 3 Supplementary Figures

**Supplementary Figure S1.** Propagation systems, rooting media preparation, and long-term cutting responses of *A. formosana*. (A) Preparation of the rooting substrates (from left to right: Substrate A, C, B, and D). (B) Cuttings directly inserted into pre-moistened horticultural rockwool blocks (Substrate E) for continuous hydration. (C) Cuttings cultivated in transparent flexible plastic pots to facilitate visual observation. (D) Non-destructive monitoring of adventitious root emergence through the transparent pot wall, eliminating the need for physical disturbance to the cuttings. (E) Initial root development of a cutting propagated in Substrate E. (F) Late-stage mortality observed in a Substrate E cutting after over 11 months of propagation, demonstrating that initial adventitious rooting does not guarantee long-term survival. (G) Vigorous root system development and sustained cutting viability after 13 months of propagation under the optimal treatment combination (Substrate A + IBA).

**Supplementary Figure S2.** Pearson correlation heatmap of maternal morphological traits in *A. formosana*. The matrix displays pairwise correlation coefficients (*r*) among diameter at breast height (DBH), basal diameter (BD), and plant height for 33 donor trees. All pairwise correlations were statistically significant (*p* < 0.001): DBH × BD, *r* = 0.982; DBH × Height, *r* = 0.891; BD × Height, *r* = 0.900.

**Supplementary Figure S3.** Panorama of independent Kaplan-Meier survival curves based on maternal physiological traits, sex, and genotypic variation in *A. formosana*. All panels display cumulative survival probabilities of cuttings over the 374-day Stage 2 observation period. Log-rank test statistics (χ² and p-values) are shown directly on each panel. Panels are stratified by: (A) diameter at breast height (DBH) group; (B) plant height group; (C) plant sex (note: sex was determinable for only 6 of 33 donor trees); (D) four genotypes selected post hoc to represent the extremes of inter-genotypic survival variation (DG-15-1, DG-15-4, DG-15-6, and DG-15-9), covering the most resilient and the most susceptible survival profiles observed across all 12 management units. Basal diameter is not shown as a separate panel because its median split partitions the donor trees identically to DBH (*r* = 0.982), producing the same survival curves and log-rank statistic. Event time is the scheduled inspection date at which death was first confirmed.

**Supplementary Figure S4.** Grid plot of genotype-specific Kaplan-Meier survival curves stratified by hormone treatment in *A. formosana*. Each panel displays survival trajectories for one management unit over the 374-day Stage 2 observation period under three treatments: untreated control (CK), IBA, and NAA. Panels cover the 12 units from which cuttings were taken. No cuttings were taken from DG-15-5, DG-15-8 or DG-15-10, so these three units have no panel. Panel titles give the total number of cuttings for each unit and the legend of each panel gives the number of cuttings per treatment. Panels are ordered by decreasing sample size and share a common vertical and horizontal scale. Multivariate log-rank testing across all 12 management units indicated significant overall inter-genotypic differences in survival under IBA (*χ²* = 37.237, *p* < 0.001) and NAA (*χ²* = 52.192, *p* < 0.001) conditions, but not under CK (*χ²* = 11.904, *p* = 0.371). Complete pairwise log-rank results for all 66 genotype combinations are provided in Supplementary Table S6. Event time is the scheduled inspection date at which death was first confirmed.

**Supplementary Figure S5.** Nelson-Aalen smoothed hazard rate h(t) with pointwise 95% confidence bands for each substrate-by-PGR combination in the Stage 1 experiment. Panels are arranged by substrate (rows A to E) and by plant growth regulator (columns), and all 25 panels share a common vertical scale so that hazard magnitudes are directly comparable across cells. The number of cuttings and the number of deaths are given in each panel. The kernel bandwidth is 40 days, matching Figure 1. The bands are wide because each panel is estimated from a single treatment cell, with a median of 8 cuttings per cell and 16 of the 25 cells below 10. These curves are descriptive. Formal inference on substrate and PGR effects rests on the Cox proportional hazards model in Table 3, which pools all 252 cuttings and reports exact hazard ratios with 95% confidence intervals, and on the log-rank tests reported in the text. Deaths were recorded only at the scheduled inspection dates, so the estimated hazard describes mortality risk on that inspection grid rather than at exact times of death.

**Supplementary Data Sheet 1.** Raw survival count matrix for the Stage 1 substrate-by-plant-growth-regulator (PGR) trial in *A. formosana*. Each row represents one of 25 treatment combinations (five PGR treatments: ABT, IBA, IAA, NAA, and an untreated control CK, crossed with five substrates A–E). The Initial_N column gives the number of cuttings established per combination at the start of the trial. Subsequent columns give the number of deaths newly recorded within each of 14 monitoring intervals (30, 54, 92, 126, 191, 237, 298, 330, 353, 383, 413, 448, 494, and 516 days post-insertion). These data underlie Tables 2 and 3, Figure 1, Supplementary Tables S3–S5, and Supplementary Figure S5. The Stage 1 rows of Supplementary Table S2 and the Stage 1 panels of Supplementary Tables S7 to S9 and S11 come from the same matrix. A death counted in an interval is attributed to the inspection date that closes it, and that date is the event time used in every analysis.

**Supplementary Data Sheet 2.** Raw survival count matrix and maternal donor characteristics for the Stage 2 genotype-by-auxin trial in *A. formosana*. Each row represents one maternal donor tree, identified by a unique code and genotype (DG-15-1 to DG-15-15). Donor attributes include spatial coordinates (X-67, Y-67), diameter at breast height (DBH, cm), basal diameter (cm), tree height (m), and sex (where determinable). The Initial_N columns give the number of cuttings established per donor under each of three treatments (IBA, NAA, and untreated control CK) at the start of the trial. Subsequent grouped columns give the number of deaths newly recorded within each of 11 monitoring intervals (58, 80, 108, 156, 181, 211, 241, 271, 315, 352, and 374 days post-insertion) for each treatment. These data underlie Table 4, Figures 2–4, Supplementary Tables S1, S6 and S7, and Supplementary Figures S2–S4. The Stage 2 rows of Supplementary Table S2 and the Stage 2 panels of Supplementary Tables S8 to S11 come from the same matrix. A death counted in an interval is attributed to the inspection date that closes it, and that date is the event time used in every analysis.
